# Genetic Algorithm-Driven Optimization of Mixed-Strain Fermentation for Improving the Physicochemical, Antioxidant, and Sensory Properties of Wampee (*Clausena lansium* (Lour.) Skeels) Juice

**DOI:** 10.3390/foods14234001

**Published:** 2025-11-22

**Authors:** Xianquan Zhong, Lin Zhang, Rong Liu, Hua Chen, Zhiheng Zhao, Xiaonuo Li, Kun Cai, Weimin Zhang, Xiaoping Hu, Xue Lin

**Affiliations:** 1 School of Food Science and Engineering, Hainan University, Haikou 570228, China; 23210832000023@hainanu.edu.cn (X.Z.); linzhang@hainanu.edu.cn (L.Z.); chenh330@163.com (H.C.); 21210832000034@hainanu.edu.cn (Z.Z.); qwaslixiao@foxmail.com (X.L.); caikun@hainanu.edu.cn (K.C.); zhwm1979@163.com (W.Z.); 2 Key Laboratory of Food Nutrition and Functional Food of Hainan Province, Haikou 570228, China; 3 Department of Nutrition and Health, Key Laboratory of Functional Dairy, China Agricultural University, Beijing 100083, China; liurong@cau.edu.cn

**Keywords:** wampee juice, lactic acid bacteria, mixed fermentation, genetic algorithm, volatile compounds, physicochemical and sensory properties

## Abstract

The potential of lactic acid bacteria (LAB) to enhance fruit juice is well known; however, the optimal fermentation strategy for wampee juice (WJ), considering its physicochemical and sensory characteristics and antioxidant activity, remains to be explored. For WJ fermentation, a co-culture of three LAB strains—*Pediococcus pentosaceus* SL05, *Pediococcus acidilactici* SL08, and *Lactiplantibacillus plantarum* JYLP-002—was optimized using a combined approach of simple lattice mixture design and a genetic algorithm (GA). After 48 h of fermentation, the optimal mixed fermentation group demonstrated a comprehensive superiority over both the unfermented and single-strain fermentation groups. It exhibited significantly higher levels of viable bacterial counts, total organic acid content, total phenolic and flavonoid contents, antioxidant capacity, and an enriched profile of volatile flavor compounds. Moreover, comprehensive volatile profiling via HS-SPME-GC-MS and HS-GC-IMS identified 59 and 29 volatile components, respectively; *β*-phellandrene, *α*-terpineol, *α*-pinene, methyl acetate, (E)-3-hexen-1-ol, and 3-methyl-1-butanol, as well as butanal, (E)-3-hexenoic acid, *β*-pinene, and propyl butanoate, were considered as key aroma contributors in the fermented WJ (FWJ). This study provides a theoretical foundation and practical framework for the enhancement of WJ quality through mixed LAB fermentation.

## 1. Introduction

Wampee (*Clausena lansium* (Lour.) Skeels), a fruit native to southern China and belonging to the Rutaceae family, is a rich source of bioactive compounds, including sesquiterpenes [1], alkaloids [2], coumarins [3], and volatile oils [4]. Fresh wampee is highly perishable and is primarily consumed as fresh fruit or juice. Although the peel of fully matured wampee is edible [5], its pronounced astringency often compromises sensory acceptability, leading to its frequent disposal as waste and limiting its reasonable utilization in the process. Furthermore, fresh wampee juice (WJ) suffers from progressive deterioration of its color, flavor, and nutritional composition during storage. Notably, the fruit’s high content of reducing sugars makes it an excellent substrate for fermentation. Therefore, new approaches to conventional processing techniques, particularly lactic acid bacteria (LAB) fermentation, are being explored to preserve or enhance the bioactivity and flavor of wampee, while simultaneously improving its storage stability and extending its shelf life.

LAB are pivotal probiotic microorganisms, and their application in fruits and vegetable fermentation is widely recognized as a health-promoting technology. This process has been shown to improve the bioactive components, functional properties, and sensory characteristics of the final products [6]. For instance, fermentation of carrot pulp with *Lactiplantibacillus plantarum* and *Pediococcus pentosaceus* significantly increased the content of total phenols and flavonoids, thereby boosting the antioxidant activity of the product [7]. LAB secrete a variety of enzymes, such as *β*-glucosidase, glycosyl hydrolases, and esterases, which facilitate the breakdown of polyphenolic compounds into simpler forms or the hydrolysis of bound phenolic acids to free phenols, thereby increasing their bioavailability [8]. Concurrently, the metabolic activities of LAB generate a range of volatile components, which are crucial in shaping the final flavor profile [9]. Generally, each LAB strain possesses a distinct metabolic profile, suggesting that the selection or combination of specific strains can be used to tailor the flavor and functional attributes of fermented products [10].

Mixed-culture fermentation, involving both synergistic and antagonistic microbial interactions, offers a more complex system than monoculture. This approach can not only greatly shorten fermentation time but also enhance the product’s complexity, texture, and flavor through the generation of a broader spectrum of metabolites, including acids, alcohols, aldehydes, and esters [11]. The efficacy of this strategy is well-documented across various fruits. For example, mixed LAB fermentation of black mulberry juice enhanced metabolic diversity and optimized flavor while reducing bitterness and astringency and minimizing the loss of vitamins [12]. Co-fermentation in apple juice enhanced antioxidant activity, improved aroma and flavor, and increased overall sensory acceptability [13]. Orange wine fermented with *Lactiplantibacillus plantarum* BC114 showed comprehensive improvements in volatile complexity, total phenolic and flavonoid contents, antioxidant capacity, coloration, palate attributes, and overall sensory evaluation scores [14]. Therefore, the selection and combination of LAB strains is vital for developing premium fermented fruit juice products. However, despite the clear potential of LAB fermentation to enhance the sensory and functional attributes of WJ, comprehensive studies in this area remain limited.

This study aimed to optimize the combination of LAB strains for WJ fermentation and to systematically evaluate the physicochemical, sensory, and antioxidant properties of both single-strain and mixed-strain fermentations. An optimal co-culture ratio was determined by integrating the polyphenol enrichment and lactic acid production ability of individual strains using a simple lattice mixture design and genetic algorithm (GA). In addition, a comprehensive comparative analysis was conducted to assess the impact of the optimal mixed-strain fermentation against single-strain fermentations and unfermented control across a range of parameters, including pH, viable cell counts, organic acid content, color, total phenolic and flavonol contents, antioxidant activity, and flavor profiles. These findings provide theoretical guidance for enhancing the flavor quality and bioactivity of WJ and industrial-scale fermentation.

## 2. Materials and Methods

### 2.1. Materials

Standards for n-alkanes (C4–C25) and organic acids, including lactic acid, acetic acid, oxalic acid, succinic acid, pyruvic acid, tartaric acid, malic acid, and citric acid, together with chromatographic-grade acetonitrile and methanol, were obtained from Sigma-Aldrich Co., Ltd. (St. Louis, MO, USA). Man, Rogosa and Sharpe (MRS) broth medium was purchased from Guangzhou Huankai Microbial Technology Co., Ltd. (Guangzhou, China). Folin–Ciocalteu phenol reagent, gallic acid, rutin, Trolox, and 2,2′-azino-bis (3-ethylbenzothiazoline-6-sulfonic acid) (ABTS) were purchased from Shanghai Yuanye Biotechnology Co., Ltd. (Shanghai, China).

### 2.2. Bacterial Strains and Growth Conditions

An initial screening was conducted on a collection of ten strains, comprising both the strains isolated from the distillers’ grains of Shanlan wine and fermented watermelon and commercial strains: *Limosilactobacillus fermentum* BZ5-7, *Limosilactobacillus fermentum* XL1-2, *Lactiplantibacillus plantarum* (*L. plantarum*) MA4, *Pediococcus pentosaceus* (*P. pentosaceus*) SL05, *Pediococcus acidilactici* (*P. acidilactici*) SL08, *Lactobacillus delbrueckii subsp. Bulgaricus* CICC 20247, *P. pentosaceus* JYRP9330, *Lactobacillus paracasei* JYPF-176, *Lactobacillus casei* 21, and *L. plantarum* JYLP-002. Selection was based on viable cell counts after 48 h fermentation in WJ, and *P. pentosaceus* SL05, *P. acidilactici* SL08, and *L. plantarum* JYLP-002 were identified as the optimal strains for subsequent experiments. *P. pentosaceus* SL05 and *P. acidilactici* SL08, isolated from the by-product of Shanlan wine, were deposited in the Laboratory of Food Biotechnology at Hainan University. *L. plantarum* JYLP-002 was obtained from Shandong Zhongke-Jiayi Bioengineering Co., Ltd. (Weifang, China). These strains were activated in MRS medium at 35 °C. To prepare the inoculum, the cultures were harvested by means of centrifugation (5000× *g*, 6 min, 4 °C), and the cells were resuspended in sterile 0.9% (*w*/*v*) NaCl solution. The optical density of the cell suspension at 600 nm (OD600) was measured spectrophotometrically. An OD600 of 1.0, corresponding to approximately 9 log CFU/mL, was subsequently used for the fermentation of WJ.

### 2.3. Sample Preparation

Wampee fruit was acquired from a market in Haikou, China. The seeds were removed and the juice was extracted by squeezing. The juice was subsequently filtered through a 100-mesh sieve to remove coarse fibers. The filtrate was then diluted with distilled water (1:1, *v*/*v*) and the sugar concentration was adjusted to 12°Bx. The pH was adjusted to 4.50 with 10% (*w*/*v*) NaHCO_3_ solution. The WJ was pasteurized at 85 °C for 15 min in a water bath and then rapidly cooled. The WJ was inoculated with 1% (*v*/*v*) of previously prepared bacterial suspension. Fermentation was carried out at 37 °C for 48 h to produce fermented wampee juice (FWJ). As a control, uninoculated WJ was incubated under identical culture conditions.

### 2.4. Experimental Design for the Mixed Fermentation of WJ

A simplex lattice mixture design was employed to formulate the three LAB strains, *P. pentosaceus* SL05, *P. acidilactici* SL08, and *L. plantarum* JYLP-002, using Design-Expert V8.0.6.1 (Stat-Ease, Inc., Minneapolis, MN, USA). For the experimental design, total phenolic content (TPC) and lactic acid content were selected as the primary response variables. The specific experimental combinations and their corresponding response values are listed in Table S1. The data were fitted to a third-order Scheffé polynomial model [15], as described in Equation (1). The adequacy of the fitted model was assessed via analysis of variance (ANOVA).

(1) $$Y=\beta_{X_{1}}X_{1}+\beta_{X_{2}}X_{2}\;{+\beta }_{X_{3}}X_{3}+\beta_{X_{1}X_{2}}X_{1}X_{2}+\beta_{X_{1}X_{3}}X_{1}X_{3}+\beta_{X_{2}X_{3}}X_{2}X_{3}+\beta_{X_{1}X_{2}X_{3}}X_{1}X_{2}X_{3\;}$$

### 2.5. Optimization of Mixed Fermentation Models

The predictive models for TPC and lactic acid content, derived from a simplex lattice mixture design, served as fitness functions for the genetic algorithm (GA) to identify the optimal LAB strain combination [16]. Optimization was performed using the Optimization Tool in MATLAB R2018a (The MathWorks, Inc., Natick, MA, USA). A custom fitness function, named Funn1, was defined in MATLAB to interface with the GA. This function takes a vector X of strain ratios as input and returns the objective value Y from the predicted polynomial model (detailed in Section 2.4). The input variables X_1_, X_2_, and X_3_ correspond to the inoculation proportions of *P. pentosaceus* SL05, *P. acidilactici* SL08, and *L. plantarum* JYLP-002, respectively.

In the Optimization Tool, the parameter of ‘Number of variables’ was set to 3. Subsequently, ‘Aeq’ and ‘beq’ in ‘Linear equalities’ were set to [1 1 1] and 1, respectively, which mathematically enforces the condition that the sum of the three strain proportions must equal 1. ‘Plot functions’ were configured to ’best fitness’, while all other GA parameters were maintained at their default settings.

### 2.6. Microbiological and Physicochemical Analyses of FWJ

#### 2.6.1. Measurement of Viable Cell Counts of LAB

The viable LAB count in FWJ was measured by means of standard plate count. Briefly, 1 mL of FWJ was subjected to a ten-fold serial dilution series in sterile 0.9% (*w*/*v*) NaCl solution. Subsequently, 1 mL of each dilution was inoculated onto an MRS agar plate in triplicate and then incubated anaerobically at 37 °C for 48 h. Viable cell counts of LAB are expressed as log CFU/mL.

#### 2.6.2. Analyses of pH and Organic Acid Composition

FWJ was filtered through a 0.45 µm PVDF membrane (Merck Milipore Ltd., Cork, CT, Ireland), and the pH of the filtrate was determined with a calibrated pH meter (Mettler-Toledo Instrument Co., Ltd., Shanghai, China). The concentrations of organic acids in FWJ were analyzed using an Agilent 1260 high-performance liquid chromatography (HPLC) system (Agilent Technologies, Inc., Santa Clara, CA, USA) equipped with a ZORBAX SB-Aq column (5 μm, 4.6 mm × 250 mm, Agilent Technologies, Inc., CA, USA). Detection was carried out at 210 nm using a UV-visible spectrum. The concentration of each organic acid was quantified with its external calibration curve.

#### 2.6.3. Determination of Color Indices

The color of WJ before and after fermentation was assessed using a TS4020 benchtop spectrophotometer (Guangdong Threenh Technology Co., Ltd., Shenzhen, China). The instrument was first calibrated with distilled water as the white reference and air as the black reference. Subsequently, an empty cuvette was positioned in the optical path to record the baseline. Finally, the samples filtered through 0.45 µm PVDF membranes were transferred into the cuvette, and the colorimetric absorbances were measured.

### 2.7. Analyses of Total Polyphenols Content (TPC) and Total Flavonoid Content (TFC) in FWJ

Referring to the protocol of Zhou et al. [17] with some modifications, the TPC was determined. Briefly, 100 μL of the diluted sample was mixed with 50 μL of Folin–Ciocalteu reagent and incubated for 5 min. Subsequently, 300 μL of 20% (*w*/*v*) Na_2_CO_3_ solution was added, and the mixture was incubated for 8 min. The absorbance was recorded at 765 nm. Gallic acid was used as the reference, and a regression curve (Y = 0.0141X + 0.1719, R^2^ = 0.9996) was constructed.

The aluminum chloride colorimetric assay was used to quantify the TFC according to the method of Yang et al. [8], with some modifications. In brief, 100 μL of the diluted sample was mixed with an equal volume of distilled water and 50 μL of 50 mg/mL NaNO_2_ solution. The reaction mixture was allowed to stand for 6 min, and then 50 μL of 100 mg/mL AlCl_3_ was added; after a further 6 min, 300 μL of distilled water and 400 μL of 1 M NaOH were introduced. After 15 min incubation, absorbance was recorded at 510 nm. A regression curve (Y = 0.0009X + 0.0184, R^2^ = 0.9991) was employed to determine TFC, using gallic acid as the reference.

### 2.8. Antioxidant Activity Analysis of FWJ

ABTS radical scavenging activity was assessed according to the method reported by Shi et al. [18], with minor modifications. In short, 100 μL of sample was mixed with 800 μL of freshly prepared ABTS·^+^ solution; the resulting mixture was then incubated at 25 °C for 30 min under illumination. Subsequently, the absorbance at 734 nm was measured. Moreover, a regression curve (Y = −0.0004X + 0.6865, R^2^ = 0.9994) was established using a series of Trolox standards (0, 20, 40, 80, 100, 150, 200, 300, and 600 µM). The results were presented as μg Trolox equivalents (TE)/mL.

The cupric-reducing antioxidant capacity (CUPRAC) assay was performed following the method of Borahan et al. [19]. Firstly, 10 μL of the sample was mixed with 100 μL of 5 mM CuSO_4_, 100 μL of neocuproine (3.75 mM), and 100 μL of CH_3_COONH_4_. (1 M) Subsequently, the reaction mixture was incubated at 25 °C for 30 min under illumination, after which the absorbance was measured at 450 nm. A series of Trolox standards (0, 50, 100, 150, 200, 250, and 300 µM) were prepared, from which a regression curve (Y = 0.0042X + 0.0214, R^2^ = 0.9996) was constructed. The final results are expressed as μg Trolox equivalents (TE)/mL.

### 2.9. E-Nose Analysis of FWJ

The flavor profile of each sample was determined using a Portable Electronic Nose PEN3 (AIRSENSE Analytics GmbH, Schwerin, MV, Germany). Briefly, the instrument was equilibrated for 30 min at room temperature; subsequently, 20 g of sample was transferred to a 40 mL vial and purged with air for 100 s prior to detection. The probe was then inserted into the headspace and held for 60 s.

### 2.10. E-Tongue Analysis of FWJ

The taste profile of each sample was evaluated with an Insent Taste System TS-5000Z (Intelligent Sensor Technology Inc., Atsugi, KG, Japan), quantifying sourness, bitterness, astringency, aftertaste-B, aftertaste-A, intensity, freshness, and saltiness. Each sample was centrifuged at 6000 rpm for 10 min, and 70 mL of the resulting clear supernatant was subjected to E-tongue analysis.

### 2.11. Sensory Evaluation of FWJ

Sensory evaluation of FWJ was performed by a trained panel of 10 assessors (aged 21–35) from Hainan University. The evaluation was based on a structured scoring system that assessed four key attributes: appearance, aroma, taste, and typicality. The detailed information about their grading criteria is presented in Table S3. A fuzzy comprehensive evaluation method was employed to quantify the sensory scores. The process involved three key steps. First, a comment set P is established for the sensory evaluation of FWJ.

(2) $$P=(P_{1},{\;P}_{2,}{\;P}_{3},\;P_{4})$$

P_1_, P_2_, P_3_, and P_4_ correspond to excellent, good, general, and worse, respectively; second, the evaluation weight X for the factors is obtained based on a subjective weighting method.

(3) $$X=(X_{1},{\;X}_{2},{\;X}_{3},{\;X}_{4})=(0.2,\;0.25,\;0.3,\;0.25)\;$$

Finally, sensory score Y for each sample is calculated by applying the fuzzy transformation principle, as shown in Equation (4), where R represents the normalized fuzzy relationship matrix.

(4) $$Y={\mathrm{XRP}}^{T}$$

### 2.12. HS-SPME-GC–MS Analysis of FWJ

First, 5 mL of the sample was added to a 20 mL glass vial, spiked with 20 µL of 2-octanol (1.11 mg/mL) as an internal standard. Headspace adsorption was performed for 60 min using a solid-phase microextraction (SPME) probe equipped with a DVB/CAR/PDMS fiber tip. Subsequently, the probe was immediately transferred to the inlet of the Agilent 8860-5977B gas chromatography (GC) system (Agilent Technologies, Inc., CA, USA) for thermal desorption at 240 °C for 5 min. The separation process was carried out on an HP-Innowax capillary column (30 m × 0.25 mm × 0.25 μm, Agilent Technologies, Inc., CA, USA), using helium (99.999%) as the carrier gas at a constant flow rate (1 mL/min). The injection temperature was maintained at 250 °C. The column temperature was set as follows: an initial hold at 40 °C for 1 min, a ramp to 120 °C at 3 °C/min and then a hold for 2 min, a subsequent increase to 240 °C at 5 °C/min and then a hold for 5 min, and a final increase to 250 °C and then a hold for 5 min. Mass spectrometry (MS) detection was carried out in electron ionization (EI) mode. The MS parameters were set as follows: ion source temperature, 245 °C; ionization energy, 70 eV; quadrupole temperature, 240 °C; mass scanning range, 40–600 *m*/*z*; and emission current, 35 μA. Retention indices (RI) were calculated using n-alkanes (C9–C28) as an external standard. Volatile compounds were identified by matching their mass spectra against the NIST 20 library and RI values. Quantification was achieved by comparing the peak area of each analyte to that of the internal standard.

### 2.13. HS-GC-IMS Analysis of FWJ

Volatile compounds in FWJ were characterized using a FlavourSpec^®^ GC-ion mobility spectrometry (IMS) system (G.A.S. Gesellschaft für analytische Sensorsysteme mbH, Dortmund, Germany). First, 1.0 mL of the sample was placed into a 20 mL headspace vial. The sample was incubated at 60 °C for 15 min, with the injection needle at 85 °C and agitation at 500 rpm. For headspace analysis, a 300 μL sample was automatically injected. Chromatographic separation was conducted on an MXT-WAX column (15 m × 0.53 mm × 1 μm, Shimadzu Corporation, Kyoto, Japan) held at 60 °C for 30 min. The carrier gas, high-purity nitrogen (99.999%) gas, was programmed with a flow rate gradient: initially 2 mL/min, increased to 10 mL/min and then maintained for 10 min, subsequently ramped to 100 mL/min and then held for 10 min, and finally reaching 150 mL/min and then held for 10 min. Drift tube IMS was maintained at 45 °C and nitrogen (99.999%) was employed as the drift gas at a constant flow rate of 150 mL/min. RI were calculated using n-alkanes (C4–C9) as an external reference. Volatile compounds were identified by comparing their RI and drift times with those of the standards in the reference library of GC-IMS (G.A.S. Gesellschaft für analytische Sensorsysteme mbH, Dortmund, Germany).

### 2.14. Statistical Analyses

All experiments were performed in triplicate (*n* = 3), and the results are presented as the mean ± standard deviation (SD). Statistical significance was determined using one-way analysis of variance (ANOVA) followed by Duncan’s multiple range test, using SPSS Statistics 26.0 (SPSS Inc., Chicago, IL, USA). A *p*-value < 0.05 was considered statistically significant. Multivariate statistical analyses, including principal component analysis (PCA) and orthogonal partial least squares discriminant analysis (OPLS-DA), were performed using SIMCA 14.1 (MKS Umetrics AB, Umea, MM, Sweden). Data visualization was carried out using OriginPro 10.1 (OriginLab Corporation, Northampton, MA, USA).

## 3. Results

### 3.1. Optimization of Inoculation Ratio in Mixed LAB Fermentation

A simplex lattice mixture design was employed to investigate the effect of different LAB strain ratios on TPC and lactic acid content in FWJ. After 48 h of fermentation, the maximum lactic acid concentration reached 4.22 ± 0.04 g/L and TPC reached 1.41 ± 0.02 TPC mg GAE/mL (Table S1). These two parameters were selected as optimization responses due to their critical influence on the quality and nutritional value of FWJ. During fermentation, LAB metabolize carbohydrates into lactic acid [20], and the resulting organic acids, in turn, modulate the transformation of phenolic compounds [21]. Consistent with previous findings reported by Gao et al. [22], LAB fermentation of individual strains and their mixtures induced lactic acid accumulation and increased TPC. Notably, mixed-strain fermentations yielded a greater increase in TPC than single-strain fermentations, indicating that the combination of LAB strains could modulate bacterial metabolism.

**Figure 1:**
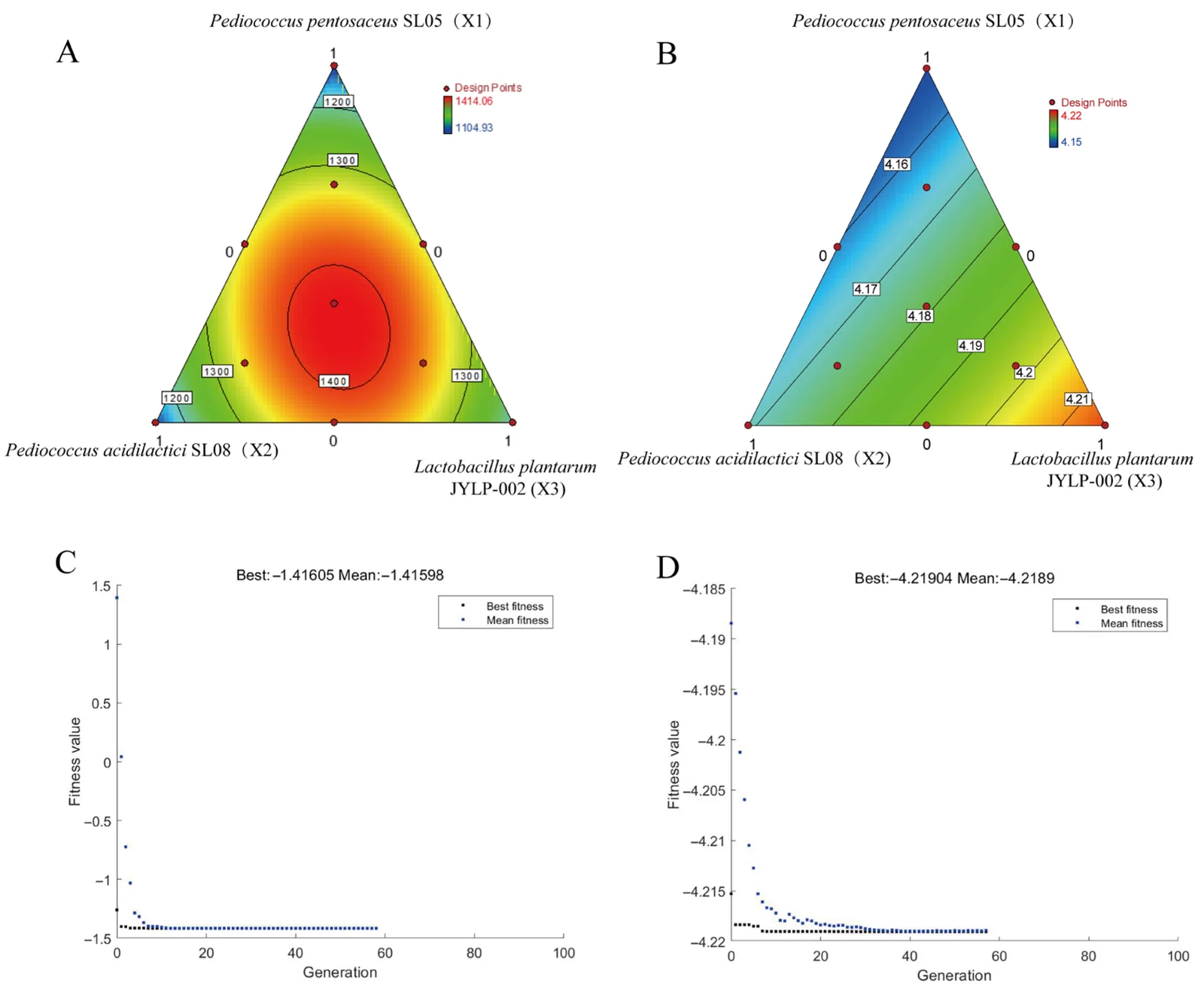
Ternary contour plot of TPC (**A**) and lactic acid content (**B**) by a simplex lattice mixture design; optimization of total phenol content (**C**) and lactic acid content (**D**) based on a genetic algorithm.

Moreover, Figure 1A presents a ternary contour plot illustrating the effect of strain ratios on TPC. It was demonstrated that there was a synergistic interaction among *P. pentosaceus* SL05, *P. acidilactici* SL08, and *L. plantarum* JYLP-002 in promoting the microbial transformation of phenolic compounds. Figure 1B reveals that a higher inoculation proportion of *L. plantarum* JYLP-002 corresponds to increased lactic acid content in FWJ.

To evaluate how different LAB strain combinations influence phenolic enrichment and lactic acid synthesis, the data in Table S1 were fitted to Equation (1). The resulting equations describing TPC (Y1) and lactic acid concentration (Y2) were as follows:

(5) $$Y_{1}\;=\;1.136X_{1}\;+\;1.217X_{2}\;+\;1.198X_{3}\;+\;0.304X_{1}X_{2}\;+\;0.524X_{1}X_{3}\;+\;0.201X_{2}X_{3}\;+\;3.100X_{1}X_{2}X_{3}$$

(6) $$Y_{2}=4.172X_{1}+4.187X_{2}+4.215X_{3}-0.023X_{1}X_{2}+0.006X_{1}X_{3}-0.029X_{2}X_{3}-0.042X_{1}X_{2}X_{3}$$

The models for TPC (R^2^ = 0.95) and the lactic acid content (R^2^ = 0.80) were both statistically significant, with model *p*-value < 0.05 and lack-of-fit *p*-value > 0.05, indicating that the predictions were reliable.

In Table S2, linear combinations of the two models with *p*-values < 0.05 indicated that each LAB strain significantly influenced both TPC and lactic acid concentration in FWJ. However, in the TPC model, the combination of *P. acidilactici* SL08 × *L. plantarum* JYLP-002 did not exert a significant effect (*p* > 0.05). Likewise, in the lactic acid model, none of the two- or three-strain LAB mixtures showed a statistically significant impact on the production of lactic acid (*p* > 0.05).

Optimization of the two regression models using a GA was conducted to identify the optimal LAB combination that can maximize the production of both TPC and lactic acid in FWJ. After 58 GA generations, the highest TPC was achieved, whereas the peak lactic acid concentration was obtained after 57 generations. The LAB mixtures of *P. pentosaceus* SL05, *P. acidilactici* SL08, and *L. plantarum* JYLP-002 inoculated at ratios of 0.33:0.31:0.36 and 0.001:0.009:0.990 yielded the maximal TPC and lactic acid content, respectively. Notably, the optimal ratios for maximizing both TPC and lactic acid, as determined via the GA (Figure 1C,D), were in strong agreement with the predictions from the simplex lattice mixture design. The collective findings presented in Figure 1A–D indicated that while the three strains exhibited synergistic biotransformation of phenolics, the combinations of *L. plantarum* JYLP-002 with the other strains did not significantly enhance lactic acid synthesis.

### 3.2. Physicochemical Characterization of FWJ Prepared via Single and Mixed LAB Fermentations

To evaluate the effects of different fermentation strategies, four experimental groups were established with an unfermented wampee juice (WJ) control. The treatments included single-strain fermentations with *P. pentosaceus* SL05 (FWJ-SL05), *P. acidilactici* SL08 (FWJ-SL08), and *L. plantarum* JYLP-002 (FWJ-002), as well as a mixed-strain fermentation (FWJ-Mix) using a co-culture of the three strains at the optimized ratio of 0.33:0.31:0.36.

#### 3.2.1. Comparison of Viable Cell Counts and pH

**Figure 2:**
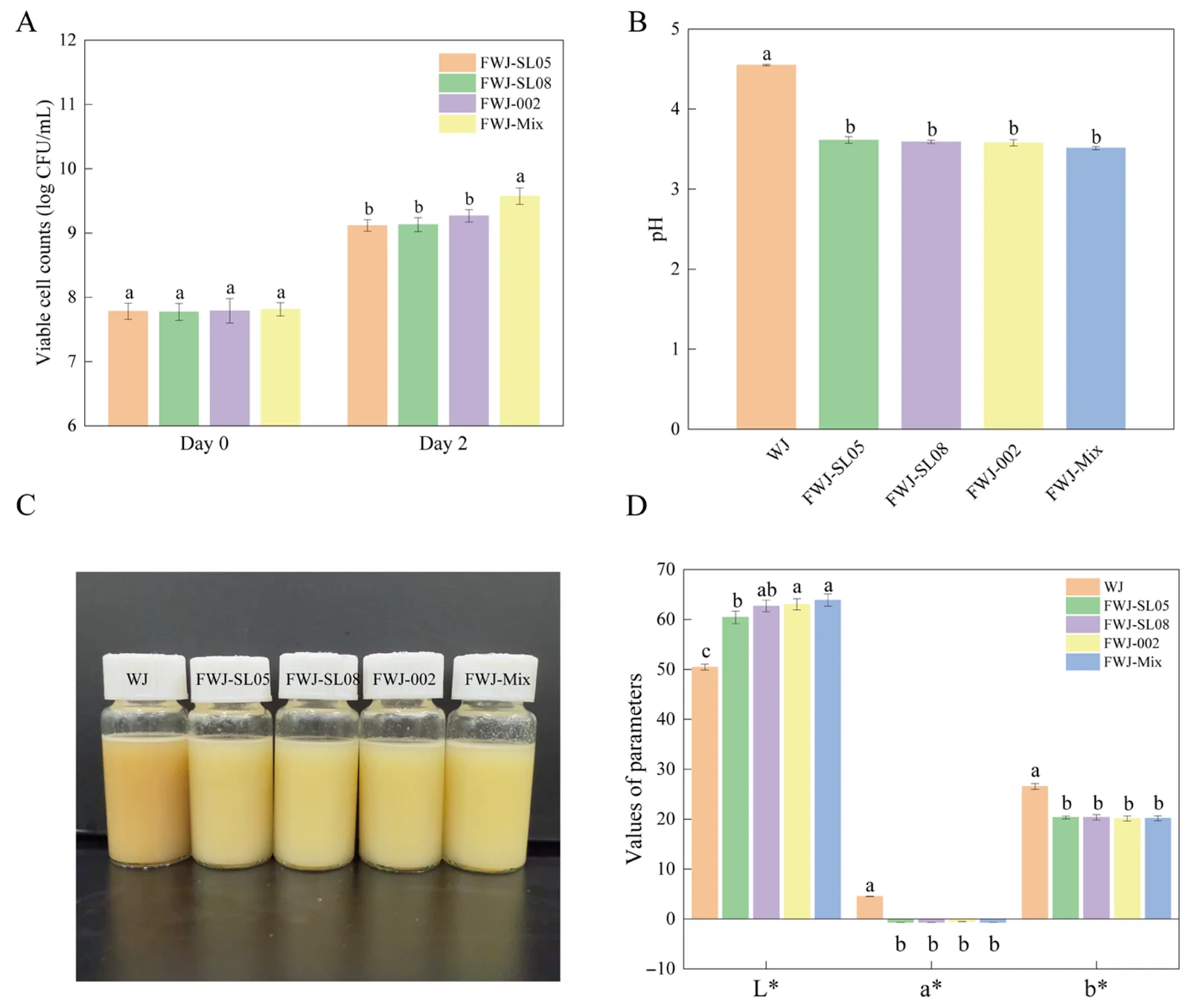
Viable cell counts (**A**) and pH (**B**) in WJ and FWJ groups; pictures (**C**) and color parameters (**D**) of WJ and FWJ samples. Bars with different lowercase letter indicate statistically significant differences (*p* < 0.05).

After 48 h of fermentation, all FWJ groups exhibited a substantial increase in viable cell counts, with the FWJ-Mix group achieving the highest population at 9.61 ± 0.07 log CFU/mL (Figure 2A). This value was significantly greater than that of all single-strain groups: FWJ-SL05 (9.21 ± 0.03 log CFU/mL), FWJ-SL08 (9.23 ± 0.02 log CFU/mL), and FWJ-002 (9.37 ± 0.02 log CFU/mL). Correspondingly, the FWJ-Mix group also had the lowest pH (3.51 ± 0.02, Figure 2B). These results demonstrated that the mix-strain culture synergistically promoted both bacterial proliferation and metabolic activity.

#### 3.2.2. Comparison of Organic Acid Composition

Organic acids are essential constituents that determine the flavor, fermentation rate, and shelf life of fermented juices [23]. As shown in Table 1, the most notable change was the emergence of lactic acid, which was undetectable in WJ but became the predominant organic acid in all FWJ samples (>4 mg/mL). This accumulation was attributed to the decarboxylation of malic acid and the homolactic fermentation of glucose [24]. The observed decrease in malic acid confirmed its role as a substrate. Furthermore, the reduction in citric acid could be due to its utilization in the tricarboxylic acid (TCA) cycle [25]. Notably, the FWJ-Mix group exhibited the highest total concentration of organic acids, indicating that mixed-strain LAB fermentation elicited a more robust metabolic activity compared to monocultures.

#### 3.2.3. Comparison of Color Indices

Color is a critical quality attribute that significantly influences consumer acceptance and serves as an important indicator for juice quality. As shown in Figure 2D, colorimetric analysis revealed that LAB fermentation significantly altered the WJ’s color profile. Compared to WJ, all FWJ samples exhibited a significant increase in L* value (lightness), coupled with significant decreases in a* (redness) and b* (yellowness) values. This trend, characterized by a lightening and reduction in chromaticity, was consistent with a previous report on LAB-fermented apple juice [26].

### 3.3. Comparison Analyses of the TPC, TFC, and Antioxidant Capacity of FWJ Prepared via Single and Mixed LAB Fermentations

LAB fermentation significantly enhanced both the TPC and TFC of WJ, as detailed in Table 1. The initial TPC of unfermented WJ was 847.23 ± 14.9 mg GAE/L; an obvious increase was observed across all samples after 48 h of fermentation. This augmentation was likely attributed to the activity of bacterial hydrolases, which liberate simpler phenolic compounds from their more complex, bound forms [26]. Given that antioxidant activity is a critical quality parameter influencing shelf life and potential health benefits, its enhancement was a key objective. As shown in Table 1, LAB fermentation significantly boosted the antioxidant activity of WJ. Notably, the FWJ-Mix sample, consistent with the results of GA optimization, exhibited the highest ABTS radical scavenging capacity (1.54 ± 0.08 μmol TE/mL) and CUPRAC reducing power (3.97 ± 0.11 μmol TE/mL), surpassing all single-strain fermentations. This observation aligned with previous research by Quan et al. [27], who reported increased antioxidant activity in LAB-fermented orange juice. Similarly, Meng et al. [28] drew an analogous finding on loquat juice fermented with a co-culture of *L. plantarum* and *Lactobacillus acidophilus*. The superior performance of the mixed-strain system can be explained by the multiple enzymes, particularly glucosidase and esterase. These enzymes can hydrolyze conjugated and bound polyphenols into simpler, free-form polyphenols, markedly increasing bioavailable polyphenol [29]. This enzymatic transformation can provide a mechanistic basis for the evaluation of TPC and the superior antioxidant capacity of the co-cultivation system relative to single-strain fermentations.

### 3.4. Sensory Evaluation of FWJ Prepared via Single and Mixed LAB Fermentations

Fuzzy comprehensive evaluation is recognized for its ability to mathematically handle ambiguity and can be used for quantifying the sensory attributes of food products [30]. By converting qualitative panel ratings into quantitative membership functions, this approach mitigates common biases, such as the halo effect, and reduces the susceptibility of results to assessor experience and inconsistency [31,32]. Consequently, it provides a robust and objective framework for integrating qualitative sensory data into a comprehensive quantitative score, making it an effective tool for evaluating both traditional and innovative food products [33].

To objectively quantify the sensory attributes of FWJ samples, a fuzzy comprehensive evaluation was employed. This approach begins by converting the qualitative panel ratings for each attribute into a rating vector. For instance, for the appearance of FWJ-SL05 (Table S4), 7 out of 10 evaluators assigned an “excellent” grade, 1 evaluator assigned “good”, 2 evaluators assigned “average”, and none assigned a “worse” grade. The appearance ratings were converted into the rating vector R Appearance = (0.7, 0.2, 0.1, 0.0). Similarly, fuzzy vectors for aroma, taste, and typicality were determined to be R Aroma = (0.8, 0.2, 0.0, 0.0), R Taste = (0.7, 0.1, 0.1, 0.1), and R Typicality = (0.6, 0.2, 0.2, 0.0), respectively. These vectors were then compiled into a comprehensive evaluation R matrix, which was employed to calculate the final sensory score (Y), according to Equation (4). The evaluation score of FWJ-SL05 was determined as follows:

(7) $$Y={\mathrm{XRP}}^{T}=(0.2,\;0.25,\;0.3,\;0.25)\times \left(\begin{array}{c} 0.7\;\;\\ 0.8\;\;\\ 0.7\;\;\\ 0.6\;\; \end{array}\begin{array}{c} 0.2\\ 0.2\\ 0.1\\ 0.2 \end{array}\begin{array}{c} 0.1\\ \;\;0.0\;\;\\ 0.1\\ 0.2 \end{array}\begin{array}{c} 0.0\\ 0.0\\ 0.1\\ 0.0 \end{array}\right)\;\times \;\left(\begin{array}{c} 100\\ 75\\ 50\\ 25 \end{array}\right)=88.5$$

The comprehensive evaluation scores of all samples are presented in Table 1. All FWJ samples achieved high scores (≥85), indicating strong consumer acceptance. Notably, the FWJ-Mix group received the highest score, surpassing the unfermented WJ, with the score of 81.88.

### 3.5. Flavor Profile Characterization of FWJ Prepared via Single and Mixed LAB Fermentations

**Figure 3:**
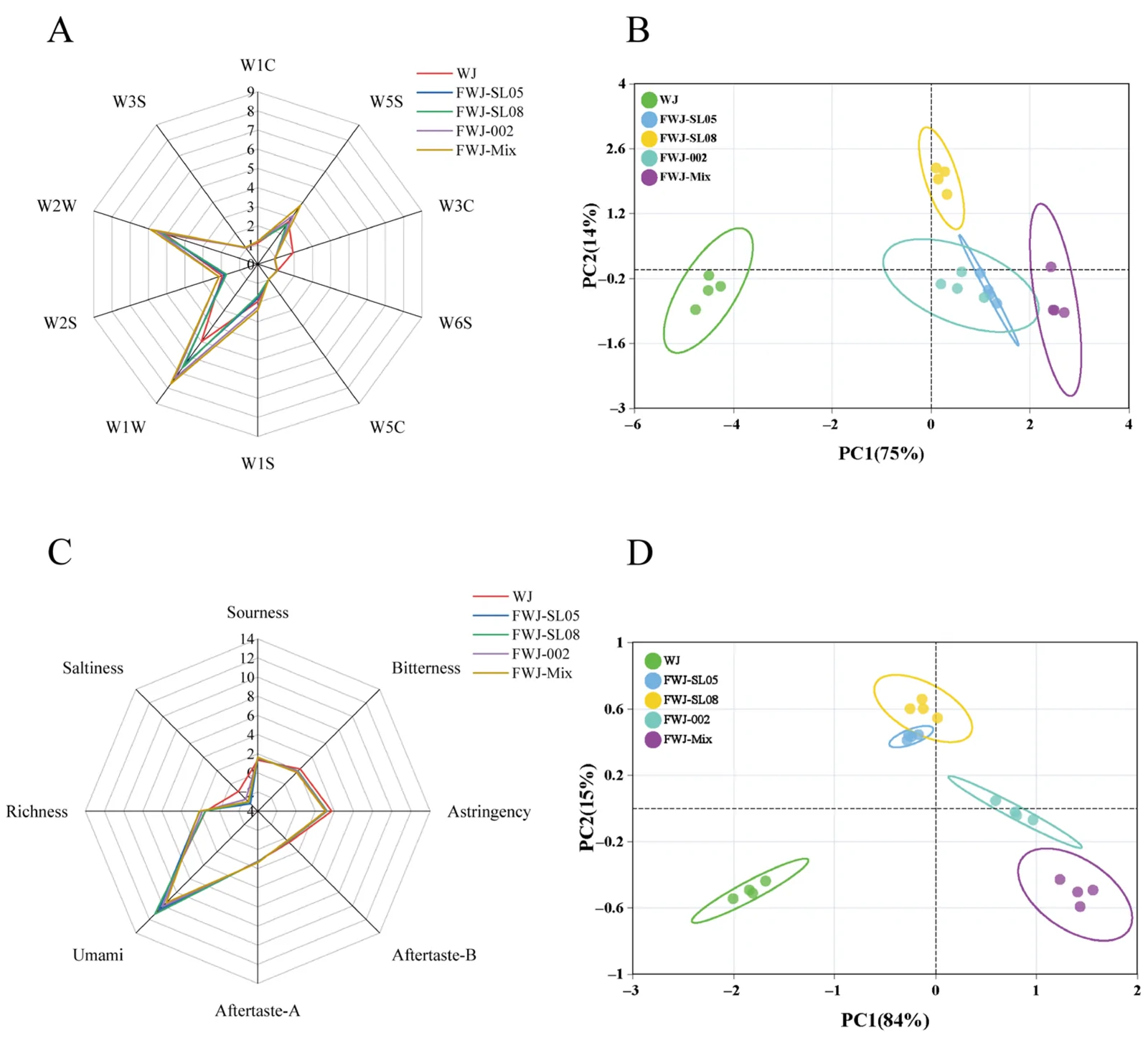
Radargram (**A**) and score plot of PCA (**B**) of WJ and FWJ samples detected using the E-nose; radargram (**C**) and score plot of PCA (**D**) of WJ and FWJ samples detected using the E-tongue.

Electronic nose (E-nose) analysis was used to characterize the volatile profiles of the samples. The radar plot of the sensor responses (Figure 3A) revealed that while the WJ and FWJ samples shared a general aromatic profile, they differed significantly. Specifically, all FWJ groups elicited markedly stronger responses for sensors W1W (inorganic sulfides) and W2S (alcohols/aldehydes/ketones). Across all sensors, the response values were higher in the FWJ samples than in the WJ control. Furthermore, the FWJ-Mix sample consistently exhibited the most intense response, indicating the greatest concentration of volatile compounds.

To further resolve the differences among samples, PCA was performed (Figure 3B). The model accounted for 66% of the total variance, with PC1 and PC2 contributing 45% and 21%, respectively. The score plot provided a clear visual discrimination of groups. It confirmed that FWJ-SL05 and FWJ-002 had similar flavors, as evidenced by their close clustering. In contrast, the WJ group was positioned farthest from the FWJ-Mix group, reflecting the most pronounced changes in the volatile profile induced by the mixed-strain LAB fermentation.

### 3.6. Taste Profile Characterization of FWJ Prepared via Single and Mixed LAB Fermentations

The flavor profiles of the samples were characterized using an electronic tongue (E-tongue), with the sensor responses visualized in a radar plot (Figure 3C). The overall flavor profiles exhibited a similar pattern, with approximate scores for aftertaste-A and aftertaste-B. Relative to WJ, LAB fermentation induced a significant shift in the taste matrix. It pronouncedly reduced bitterness, astringency, aftertaste-B and saltiness, while concurrently enhancing sourness and richness. Moreover, the sourness levels showed the following ranking: FWJ-Mix > FWJ-002 > FWJ-SL08 > FWJ-SL05 > WJ—a trend that was consistent with the total organic acid content. This modulation of flavor can be attributed to the changes in the concentrations of taste compounds. For instance, bitterness, astringency, and aftertaste-B were associated with phenolic and organic acid levels [34], while the increase in lactic acid likely masked bitterness and astringency and further intensified the perception of sourness [35]. In summary, LAB fermentation profoundly modulated the flavor of WJ.

To further discriminate the samples, PCA was performed on the E-tongue data. The model was highly robust, with the first two principal components (PC1 and PC2), collectively explaining 99% of the total variance (84% and 15%, Figure 3D). A clear separation was observed along PC1, which primarily distinguished the WJ group from all FWJ groups. Notably, samples of FWJ-SL08 were positioned farthest from samples of the other FWJ groups, indicating that this single-strain fermentation produced a distinct and unique taste profile.

### 3.7. Analysis of Volatile Components in FWJ Prepared via Single and Mixed LAB Fermentations

GC-MS is highly effective for identifying larger molecules (C10–C15), whereas GC-IMS offers superior sensitivity for smaller compounds (C2–C11) [36]. To utilize the complementary strengths of these two techniques, a combined analysis was performed, leading to the identification of 87 distinct volatile organic compounds (VOCs). As shown in Table 2, fermentation significantly enriched the volatile profile. The unfermented WJ contained 58 VOCs, while the fermented samples (FWJ-SL05, FWJ-SL08, FWJ-002, and FWJ-Mix) exhibited 70, 69, 69, and 75 VOCs, respectively.

#### 3.7.1. Qualitative and Quantitative Analysis of VOCs via HS-SPME-GC-MS

**Figure 4:**
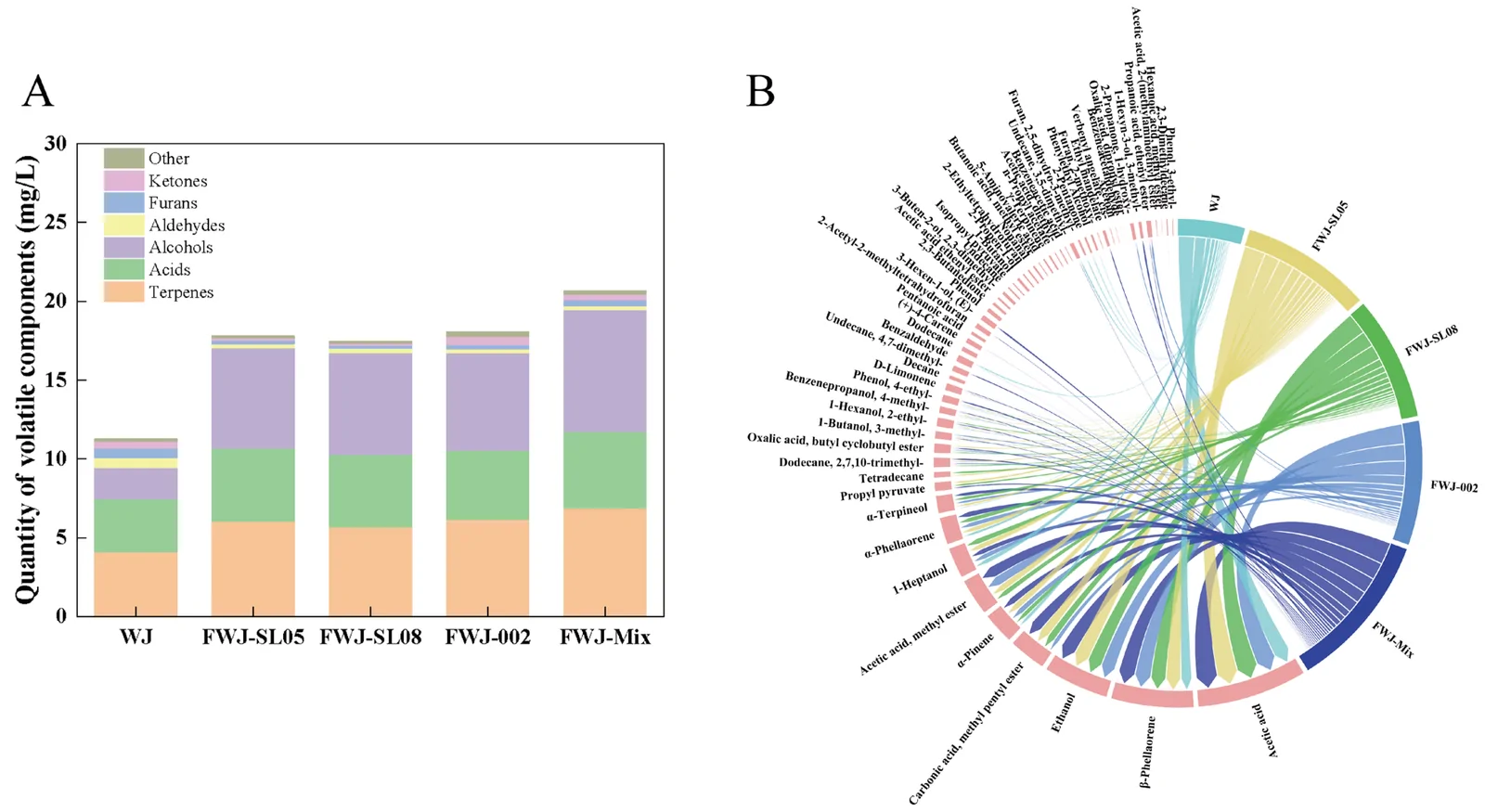
Stacked column chart for the total amount of VOCs in each category of WJ and FWJ groups detected by means of HS-SPME-GC-MS (**A**); chord diagram of the proportion of VOCs in each group (**B**).

As illustrated in Figure 4A, fermentation induced a significant enhancement in the total content of VOCs of WJ. The total VOC concentration reached 22.53, 21.33, 22.01, and 27.58 mg/L for FWJ-SL05, FWJ-SL08, FWJ-002, and FWJ-Mix, respectively. Thus, all FWJ samples exhibited marked increases in both the diversity and abundance of VOCs, compared with WJ (Table 2). The newly formed or increased VOCs were predominantly aromatic, imparting desirable fruity, green, and sweet notes (Table 2 and Table S5). These findings suggested that LAB fermentation, especially the mixed-strains approach, substantially elevated the aromatic complexity and overall quality of FWJ.

**Table 2:** Analysis of VOCs in WJ and FWJ prepared by single and mixed LAB fermentations by HS-SPME-GC-MS.

Category	RI_1_	RI_2_	VOCs	Formula	Content (mg/L)				
					WJ	FWJ-SL05	FWJ-SL08	FWJ-002	FWJ-Mix
Aldehydes	1371	1382	Nonanal	C_9_H_18_O	0.19 ± 0.01 ^a^	0.06 ± 0.00 ^b^	0.03 ± 0.00 ^b^	0.04 ± 0.00 ^b^	0.01 ± 0.00 ^b^
	1661	1662	Benzeneacetaldehyde	C_8_H_8_O	0.02 ± 0.00 ^a^	ND	ND	ND	ND
	1527	1541	Benzaldehyde	C_7_H_6_O	0.42 ± 0.02 ^a^	0.20 ± 0.01 ^b^	0.21 ± 0.01 ^b^	0.19 ± 0.01 ^b^	0.24 ± 0.01 ^b^
Terpenes	1172	1193	D-Limonene	C_10_H_16_	0.20 ± 0.01 ^a^	0.25 ± 0.01 ^a^	0.22 ± 0.01 ^a^	0.29 ± 0.01 ^b^	0.34 ± 0.01 ^b^
	1254	1253	*γ*-Terpinene	C_10_H_16_	0.03 ± 0.00 ^b^	0.04 ± 0.00 ^b^	0.04 ± 0.00 ^b^	0.04 ± 0.00 ^b^	0.19 ± 0.01 ^a^
	1199	1206	*β*-Phellandrene	C_10_H_16_	2.32 ± 0.13 ^b^	3.16 ± 0.13 ^a^	3.18 ± 0.13 ^a^	3.37 ± 0.13 ^a^	3.45 ± 0.14 ^a^
	1004	1020	*α*-Pinene	C_10_H_16_	0.83 ± 0.05 ^d^	1.39 ± 0.06 ^a^	1.01 ± 0.04 ^c^	1.23 ± 0.05 ^b^	1.45 ± 0.06 ^a^
	1141	1160	*α*-Phellandrene	C_10_H_16_	0.68 ± 0.04 ^c^	1.03 ± 0.04 ^b^	1.06 ± 0.04 ^b^	1.06 ± 0.04 ^b^	1.22 ± 0.05 ^a^
	1139	-	(+)-4-Carene	C_10_H_16_	0.10 ± 0.01 ^c^	0.18 ± 0.01 ^b^	0.17 ± 0.01 ^b^	0.19 ± 0.01 ^b^	0.25 ± 0.01 ^a^
Ketones	999	1006	2-Pentanone	C_5_H_10_O	0.36 ± 0.02 ^a^	ND	ND	ND	ND
	961	963	2,3-Butanedione	C_4_H_6_O_2_	ND	0.13 ± 0.01 ^a^	0.12 ± 0.00 ^a^	0.11 ± 0.00 ^a^	0.13 ± 0.01 ^a^
	1341	1318	2-Propanone, 1-hydroxy-	C_3_H_6_O_2_	ND	ND	ND	0.41 ± 0.02 ^a^	0.23 ± 0.01 ^b^
	1300	1296	Acetoin	C_3_H_6_O	0.06 ± 0.00 ^a^	ND	ND	ND	ND
Alcohols	1896	1907	Phenylethyl Alcohol	C_8_H_10_O	0.3 ± 0.02 ^a^	ND	ND	0.1 ± 0.00 ^b^	ND
	912	930	Ethanol	C_2_H_6_O	ND	3.11 ± 0.12 ^a^	3.02 ± 0.12 ^a^	2.94 ± 0.12 ^a^	3.18 ± 0.13 ^a^
	1387	1388	3-Hexen-1-ol, (E)-	C_6_H_12_O	ND	0.15 ± 0.01 ^b^	0.17 ± 0.01 ^b^	0.12 ± 0.00 ^b^	0.56 ± 0.02 ^a^
	1187	1119	2-Propen-1-ol	C_3_H_6_O	ND	0.08 ± 0.00 ^a^	0.07 ± 0.00 ^a^	0.02 ± 0.00 ^b^	0.09 ± 0.00 ^a^
	2197	-	Benzenepropanol, 4-methyl-	C_10_H_14_O	ND	0.32 ± 0.01 ^a^	0.27 ± 0.01 ^a^	0.29 ± 0.01 ^a^	0.36 ± 0.01 ^a^
	1507	1490	1-Hexanol, 2-ethyl-	C_8_H_18_O	0.24 ± 0.01 ^b^	0.35 ± 0.01 ^a^	0.39 ± 0.02 ^a^	0.28 ± 0.01 ^b^	0.38 ± 0.02 ^a^
	1479	1463	1-Heptanol	C_7_H_16_O	1.06 ± 0.06 ^b^	1.17 ± 0.05 ^a^	1.26 ± 0.05 ^a^	1.09 ± 0.04 ^b^	1.13 ± 0.05 ^b^
	1197	1206	1-Butanol, 3-methyl-	C_5_H_12_O	ND	0.36 ± 0.01 ^b^	0.27 ± 0.01 ^c^	0.43 ± 0.02 ^a^	0.34 ± 0.01 ^b^
	1123	1135	1-Butanol	C_4_H_10_O	ND	0.09 ± 0.00 ^b^	0.11 ± 0.00 ^a^	0.14 ± 0.01 ^a^	0.15 ± 0.01 ^a^
	1174	-	3-Buten-2-ol, 2,3-dimethyl-	C_6_H_12_O	0.07 ± 0.00 ^b^	0.11 ± 0.00 ^a^	0.12 ± 0.00 ^a^	0.14 ± 0.01 ^a^	0.13 ± 0.01 ^a^
	1369	-	1-Hexyn-3-ol, 3-methyl-	C_7_H_12_O	ND	ND	ND	ND	0.56 ± 0.02 ^a^
	1687	1683	*α*-Terpineol	C_10_H_18_O	0.31 ± 0.02 ^c^	0.63 ± 0.03 ^b^	0.79 ± 0.03 ^a^	0.66 ± 0.03 ^b^	0.84 ± 0.03 ^a^
Esters	1908	-	Verbenyl angelate, cis-	C_15_H_22_O_2_	0.07 ± 0 ^a^	ND	ND	0.03 ± 0.00 ^b^	0.02 ± 0.00 ^b^
	1373	-	Propyl pyruvate	C_6_H_10_O_3_	ND	0.63 ± 0.03 ^a^	0.55 ± 0.02 ^b^	ND	0.44 ± 0.02 ^c^
	1366	-	Carbonic acid, methyl pentyl ester	C_7_H_14_O_3_	ND	1.97 ± 0.08 ^b^	1.59 ± 0.06 ^c^	0.98 ± 0.04 ^d^	2.43 ± 0.10 ^a^
	1111	-	Propanoic acid, ethenyl ester	C_5_H_8_O2	ND	ND	ND	0.56 ± 0.02 ^a^	0.31 ± 0.01 ^b^
	894	834	Acetic acid, methyl ester	C_3_H_6_O_2_	ND	1.39 ± 0.06 ^c^	1.16 ± 0.05 ^d^	1.98 ± 0.08 ^b^	2.37 ± 0.09 ^a^
	1782	-	Ethyl mandelate	C_10_H_12_O_3_	0.11 ± 0.01 ^b^	ND	ND	ND	0.43 ± 0.02 ^a^
	1494	-	Acetic acid, 2-(methylaminoethyl) ester	C_5_H_11_NO_2_	ND	ND	ND	ND	0.13 ± 0.01 ^a^
	918	-	Acetic acid ethenyl ester	C_4_H_6_O_2_	ND	0.13 ± 0.01 ^a^	0.11 ± 0.00 ^a^	ND	0.13 ± 0.01 ^a^
	1934	-	Oxalic acid, butyl cyclobutyl ester	C_10_H_16_O_4_	0.09 ± 0.01 ^b^	0.42 ± 0.02 ^a^	0.38 ± 0.02 ^a^	0.39 ± 0.02 ^a^	0.42 ± 0.02 ^a^
	1386	-	Isopropyl pyruvate	C_6_H_10_O_3_	ND	0.10 ± 0.00 ^a^	ND	ND	0.11 ± 0.00 ^a^
	1034	1001	Butanoic acid, methyl ester	C_5_H_10_O_2_	ND	0.05 ± 0.00 ^a^	0.03 ± 0.00 ^a^	0.03 ± 0.00 ^a^	0.05 ± 0.00 ^a^
	953	966	n-Propyl acetate	C_5_H_10_O_2_	0.03 ± 0.00 ^a^	0.04 ± 0.00 ^a^	0.05 ± 0.00 ^a^	ND	0.05 ± 0.00 ^a^
	1193	1197	Hexanoic acid, methyl ester	C_7_H_14_O_2_	ND	ND	ND	ND	0.05 ± 0.00 ^a^
	1113	-	Oxalic acid, dipropyl ester	C_8_H_14_O_4_	0.02 ± 0.00 ^a^	ND	ND	ND	ND
Acids	1454	1461	Acetic acid	C_2_H_4_O_2_	3.38 ± 0.19 ^b^	4.34 ± 0.17 ^a^	4.33 ± 0.17 ^a^	4.09 ± 0.16 ^a^	4.47 ± 0.18 ^a^
	1445	-	Acetic acid, diethyl-	C_6_H_12_O_2_	ND	0.04 ± 0.00 ^c^	0.05 ± 0.00 ^b^	0.05 ± 0.00 ^b^	0.08 ± 0.00 ^a^
	2550	2568	Benzeneacetic acid	C_8_H_8_O_2_	ND	0.01 ± 0.00 ^c^	0.03 ± 0.00 ^b^	0.03 ± 0.00 ^b^	0.06 ± 0.00 ^a^
	1728	1734	Pentanoic acid	C_5_H_10_O_2_	ND	0.18 ± 0.01 ^a^	0.12 ± 0.00 ^b^	0.11 ± 0.00 ^b^	0.14 ± 0.01 ^b^
	1768	-	5-Aminovaleric acid	C_5_H_11_NO_2_	ND	0.05 ± 0.00 ^a^	0.08 ± 0.00 ^a^	0.09 ± 0.00 ^a^	0.08 ± 0.00 ^a^
Furans	1674	-	2-Acetyl-2-methyltetrahydrofuran	C_7_H_12_O_2_	0.09 ± 0.01 ^c^	0.16 ± 0.01 ^b^	0.17 ± 0.01 ^b^	0.18 ± 0.01 ^b^	0.31 ± 0.01 ^a^
	1070	-	Furan, 2-methoxy-	C_5_H_6_O_2_	0.13 ± 0.01 ^a^	ND	ND	ND	ND
	955	-	Furan, 2,5-dihydro-3-methyl-	C_5_H_8_O	0.39 ± 0.02 ^a^	ND	ND	ND	ND
	984	-	2-Ethyltetrahydrofuran	C_6_H_12_O	ND	0.08 ± 0.00 ^a^	0.06 ± 0.00 ^a^	0.08 ± 0.00 ^a^	0.09 ± 0.00 ^a^
Hydrocarbon	1000	1000	Decane	C_10_H_22_	0.21 ± 0.01 ^a^	0.22 ± 0.01 ^a^	0.23 ± 0.01 ^a^	ND	ND
	1100	1100	Undecane	C_11_H_24_	0.1 ± 0.01 ^a^	0.1 ± 0.00 ^a^	0.1 ± 0.00 ^a^	ND	ND
	1224	-	Undecane, 4,7-dimethyl-	C_13_H_28_	ND	0.21 ± 0.01 ^b^	0.2 ± 0.01 ^b^	0.21 ± 0.01 ^b^	0.43 ± 0.04 ^a^
	1218	-	Undecane, 3,5-dimethyl-	C_13_H_28_	0.46 ± 0.03 ^a^	ND	ND	ND	0.29 ± 0.01 ^b^
	1200	1200	Dodecane	C_12_H_26_	0.20 ± 0.01 ^a^	0.20 ± 0.01 ^a^	0.20 ± 0.01 ^a^	0.12 ± 0.00 ^b^	0.11 ± 0.00 ^b^
	1329	-	Dodecane, 2,7,10-trimethyl-	C_15_H_32_	ND	0.45 ± 0.02 ^b^	0.41 ± 0.02 ^b^	0.43 ± 0.02 ^b^	0.49 ± 0.02 ^a^
	1210	-	2,3-Dimethyldecane	C_12_H_26_	ND	ND	ND	0.23 ± 0.01 ^a^	ND
	1400	1400	Tetradecane	C_14_H_30_	ND	0.47 ± 0.02 ^b^	0.57 ± 0.02 ^a^	ND	ND
Other	2012	-	Phenol, 4-ethyl-	C_8_H_10_O	ND	0.29 ± 0.01 ^b^	0.28 ± 0.01 ^b^	0.27 ± 0.01 ^b^	0.49 ± 0.02 ^a^
	2011	-	Phenol, 3-ethyl-	C_8_H_10_O	ND	ND	ND	0.13 ± 0.01 ^a^	ND
	2009	2012	Phenol	C_6_H_6_O	0.17 ± 0.01 ^a^	0.13 ± 0.01 ^b^	0.11 ± 0.00 ^b^	0.14 ± 0.01 ^a^	0.18 ± 0.01 ^a^

Note: VOCs is the abbreviation of volatile organic compounds. WJ refers to wampee juice; FWJ-SL05 refers to fermented WJ with *P. pentosaceus* SL05, FWJ-SL08 with *P. acidilactici* SL08, and FWJ-002 with *L. plantarum* JYLP-002; FWJ-mix refers to fermented WJ with the optimal LAB combination. The values in the same row with different lowercase letters indicate statistically significant differences (*p* < 0.05). RI_1_, retention index detected on HP-INNOWax capillary column; RI_2_, retention index recorded in the literature; ND, not detected.

Acetic acid was the most abundant VOC identified by means of GC-MS across all groups, as illustrated in Figure 4B. It serves multiple functions, such as imparting a fresh citrus aroma, contributing to pH reduction, and providing a balanced acidic flavor profile. The second most abundant compound, *β*-phellandrene, further enriched the aroma of FWJ samples with distinct citrus and herbal notes.

Terpenes represent a particularly noteworthy category of VOCs. Six terpenes were identified (Table 2), including the representative compound *β*-phellandrene, which contributes characteristic pine, turpentine, and resinous notes. A significant increase in the concentration of all six terpenes was observed after LAB fermentation, indicating that microbial growth stimulated the biosynthesis or release of these potent aroma compounds.

Similarly, alcohols constitute another crucial class of VOCs in FWJ. They serve a dual function: acting as solvents for enhancing the perception of other aroma substances and directly contributing sweet and refreshing flavors [37]. Furthermore, fermentation led to a substantial increase in total volatile alcohol content, with levels in the FWJ groups being 2.2–2.9 times higher than in the WJ group. This elevation is likely a result of the microbial metabolism of glucose and amino acids [38], thereby enriching the beverage’s overall aroma with fruity, floral, grassy, and honey-sweet notes.

The most dramatic transformation occurred in the ester fraction, with a total of 14 distinct esters identified. Produced via microbial enzymatic catalysis or direct esterification of alcohols with organic acids [39], esters are characterized by high volatility and low sensory thresholds, conferring sweet, fruity, and floral notes even at trace concentrations [40]. Remarkably, the total ester content in FWJ was 11.4–21.7 times greater than in WJ, leading to the development of typical sweet pineapple and citrus aromas in FWJ samples (Table S5).

As the primary metabolic products of LAB fermentation, organic acids are fundamental to the flavor profile of FWJ. At concentrations below their odor threshold, they contribute to a balanced flavor while simultaneously suppressing spoilage microorganisms and mitigating off-odors [41].

Other minor yet significant compounds, including ketones, furans, and phenols, were also detected. Ketones, formed via amino acid degradation, the Maillard reaction, and free fatty acid oxidation, contribute floral, fruity, and herbaceous aroma [42]. In addition, aldehyde concentrations decreased post-fermentation and remained low, and the reduced levels may be more acceptable to consumers [43].

#### 3.7.2. Qualitative and Quantitative Analysis of VOCs via HS-GC-IMS

**Figure 5:**
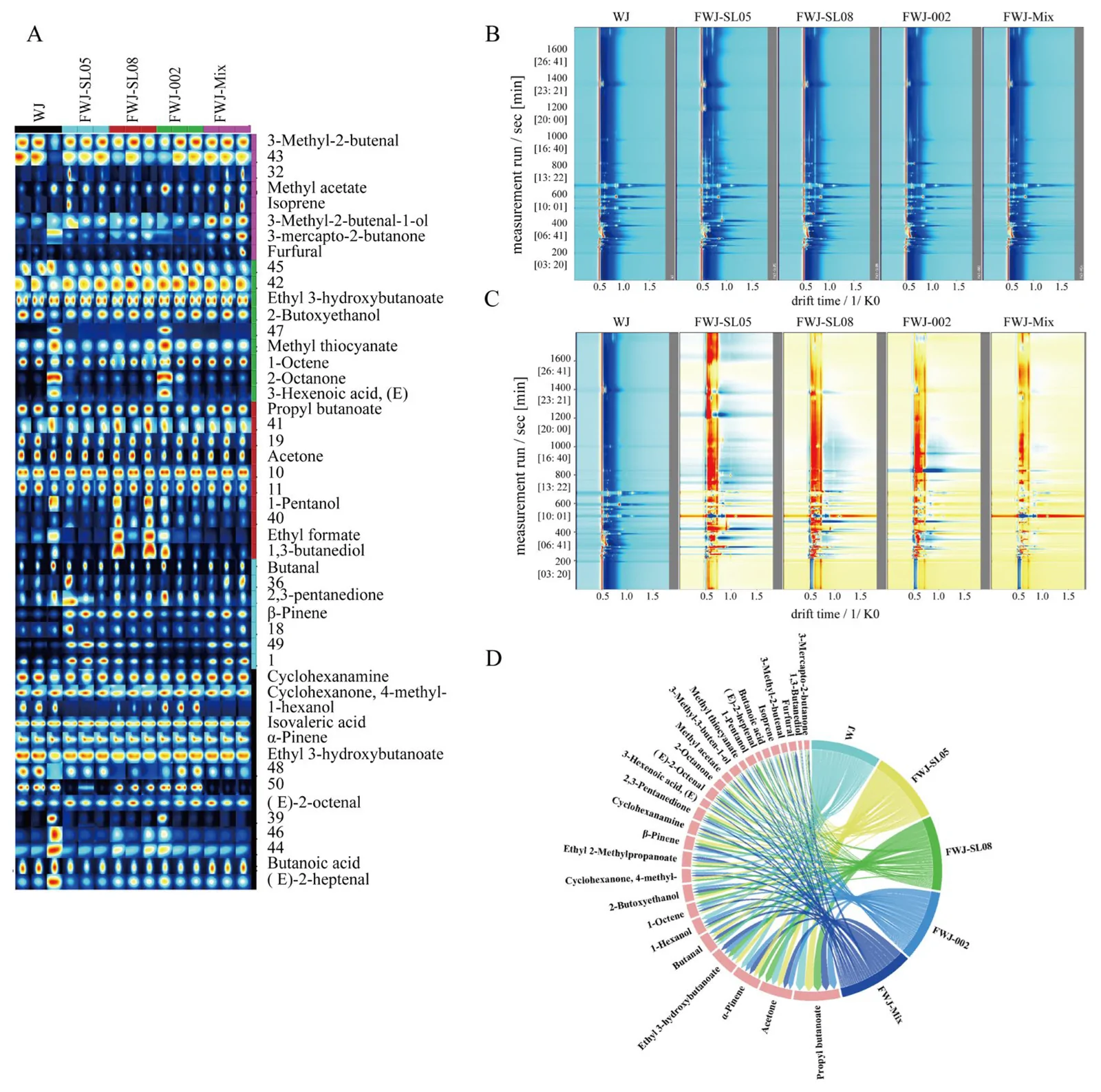
Fingerprints of VOCs (**A**), two-dimensional spectrum (**B**), and two-dimensional differential spectrum (**C**) of WJ and FWJ groups detected by means of HS-GC-IMS; chord diagram of proportion of VOCs in each group (**D**).

To visualize the overall alterations in VOCs of samples detected via GC-IMS, fingerprints were generated (Figure 5A). In the heatmap, each column represents a sample and each row corresponds to a specific VOC signal, with a total of 28 VOCs being identified. Moreover, the VOCs were clustered into five classes based on the pattern of their content change. In both Figure 5A,B, the color intensity indicated the relative concentration of each VOC, with red signifying higher abundance and blue indicating lower abundance. A more detailed comparison is presented in the differential plot (Figure 5C), which used the WJ spectrum as a reference. In this plot, white dots signify VOCs with concentrations comparable to WJ, while red and blue dots illustrate the magnitude of increase or decrease in the FWJ samples, respectively. Collectively, these visualizations confirmed that LAB fermentation profoundly altered both the composition and abundance of the VOCs. Notably, propyl butyrate was identified as having the highest relative concentration among all detected VOCs (Figure 5D), imparting characteristic pineapple and apple aromas to the FWJ.

### 3.8. OPLS-DA Analysis of VOCs in FWJ Prepared via Single and Mixed LAB Fermentations

**Figure 6:**
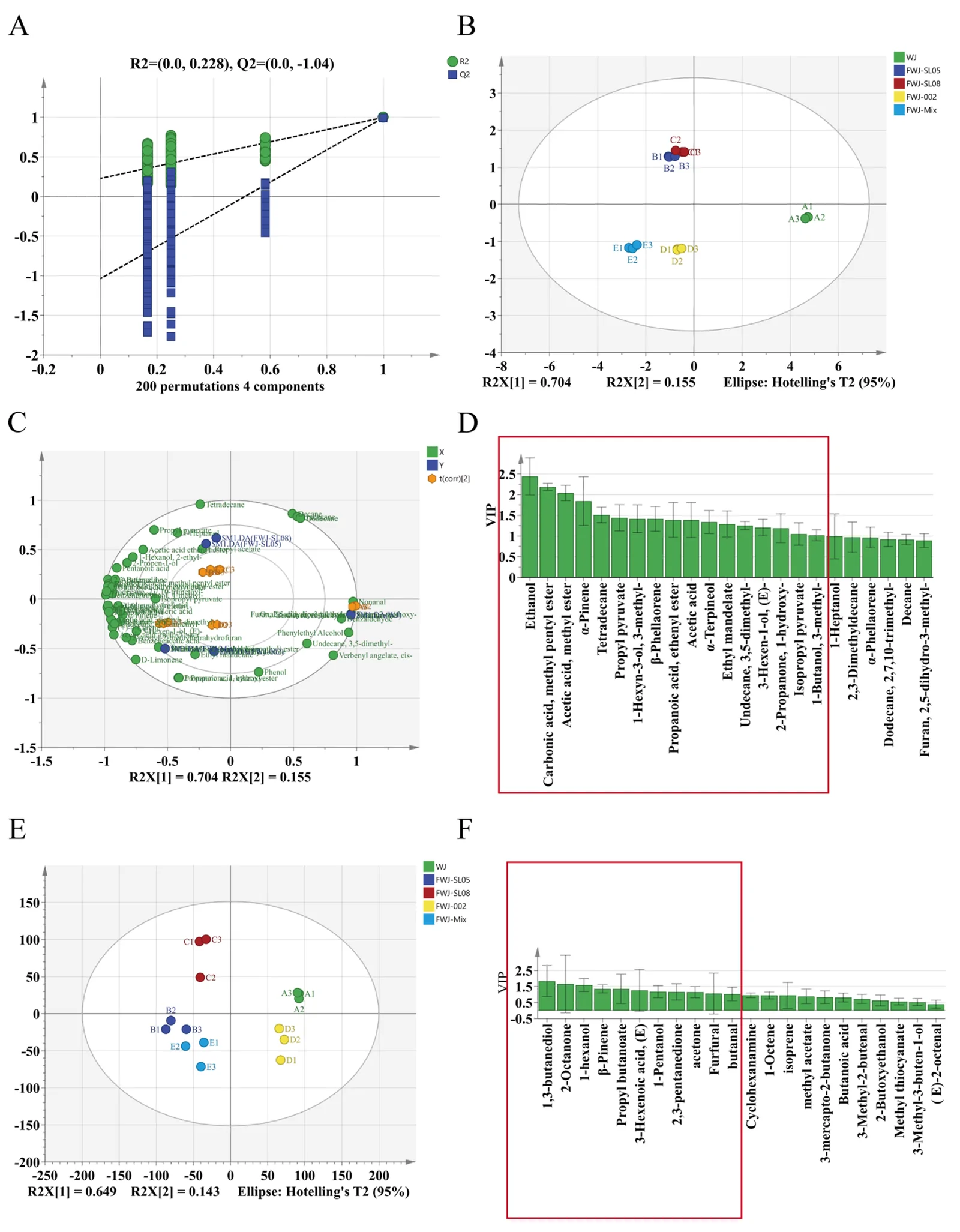
Permutation test plot (**A**), score plot (**B**), biplot (**C**) and VIP plot (**D**) of OPLS-DA for WJ and FWJ groups detected by means of HS-GC-MS; score plot (**E**); VIP plot (**F**) of OPLS-DA for WJ and FWJ groups detected by means of HS-GC-IMS. The compounds highlighted within the red box exhibit VIP > 1.

OPLS-DA, a supervised multivariate method [44], was employed for identifying the key VOCs responsible for the aroma differences among the groups. The robustness of the OPLS-DA model, based on GC-MS data, was rigorously validated through a 200-permutation test. As depicted in Figure 6A, all permuted R^2^ and Q^2^ values were lower than their original points, and the Q^2^ regression line intercepted the Y-axis below zero, confirming the model’s validity and its lack of overfitting.

Both the GC-MS and GC-IMS models demonstrated excellent explanatory ability and predictability, with the former achieving R^2^Y = 0.996 and Q^2^ = 0.988, and the latter R^2^Y = 0.965 and Q^2^ = 0.874. The corresponding score plots (Figure 6B,E) revealed a clear separation among all sample groups, confirming that each fermentation treatment produced a unique volatile fingerprint. Moreover, the specific compounds driving this separation of GC-MS measurement were identified via the biplot (Figure 6C). Specifically, the WJ group clustered in the fourth quadrant, correlating with a limited number of VOCs and consequently a simpler flavor profile. In contrast, FWJ-SL05 and FWJ-SL08 groups were positioned closely in the second quadrant, indicating similar VOC compositions. Meanwhile, FWJ-Mix and FWJ-002 occupied the third quadrant and were associated with a greater number of key VOCs, likely contributing to their enhanced flavor complexity. Similarly, as illustrated in the GC-IMS score plot (Figure 6E), FWJ-SL05 and FWJ-Mix samples clustered together in Quadrant III, indicating a similar volatile profile, while WJ, FWJ-SL08, and FWJ-002 groups occupied distinct quadrants (I, II, and IV, respectively), highlighting their unique aroma signatures.

To further elucidate the crucial contributors to these distinct profiles, key VOCs were screened using a variable importance in projection (VIP) score with a threshold greater than 1. This analysis identified 17 key VOCs from GC-MS data (Figure 6D) and 11 from GC-IMS data (Figure 6F). Collectively, these 28 compounds were considered the primary drivers of the most significant aroma changes induced by the different LAB strains.

### 3.9. Identification of Key Aroma Contributors in FWJ Prepared via Single and Mixed LAB Fermentations

To evaluate the contribution of each VOC to the overall aroma, odor activity values (OAVs) were calculated. OAVs, which integrate concentration with sensory thresholds [45], provide a quantitative measure of flavor impact. This analysis was applied to key compounds identified via both GC-MS (Table S5) and GC-IMS (Table S6). In the WJ samples, *β*-phellandrene was the dominant aroma compound (OAV = 58.08), imparting a citrusy–herbal character. Its contribution was further enhanced across all FWJ samples. After fermentation, 2,3-butanedione emerged as the most potent contributor, imparting desirable buttery, creamy, and caramel notes. Notably, several compounds, including *β*-phellandrene, *α*-terpineol, *α*-pinene, methyl acetate, (E)-3-hexen-1-ol, and 3-methyl-1-butanol, were identified as critical aroma contributors, possessing both VIP scores > 1 and OAVs > 1. In parallel, relative odor activity value (rOAV) can be employed to assess the impact of the volatile flavor compounds detected via GC-IMS [46]. As shown in Table S6, (E)-2-octenal was the primary contributor to the WJ aroma, with a fatty, green, and cucumber-like note. Most compounds exhibited a reduction in rOAV after fermentation, likely leading to a softer flavor profile of FWJ samples. However, the rOAV of *β*-pinene was enhanced, contributing a woody and piney note. Similarly, 2,3-pentanedione, butanal, (E)-3-hexenoic acid, *β*-pinene, and propyl butanoate identified via GC-IMS, were confirmed as key aroma contributors, possessing both high VIP scores and rOAVs greater than l. The synthesis of these analyses provided a clear explanation for the superior aroma profile of the FWJ-mix group. This group was characterized by significantly higher OAVs or rOAVs for its key volatiles, predominantly presenting citrusy and herbal aromas. The elevated aromatic intensity and complexity endowed the FWJ-Mix group with a more intricate and full-bodied character, distinguishing it from other samples.

## 4. Conclusions

This study systematically investigated the physicochemical, sensory and antioxidant characteristics of FWJ with both single-strain and mixed LAB. The results demonstrated that co-fermentation with a specific combination of *P. pentosaceus* SL05, *P. acidilactici* SL08 and *L. plantarum* JYLP-002 (0.33: 0.31: 0.36), which was optimized via GA, yield a product with superior levels of TPC, TFC, total organic acid, antioxidant capability compared to both the unfermented control and single-strain LAB fermentions. It is noteworthy, however, despite the optimized ratio of the three LAB strains, no synergistic interactions were observed in the synthesis of lactic acid. Furthermore, a comprehensive analysis using HS-SPME-GC-MS and HS-GC-IMS identified a complex profile of crucial volatile aroma components in the FWJ, including *β*-phellandrene, *α*-terpineol, *α*-pinene, methyl acetate, (E)-3-hexen-1-ol, 3-methyl-1-butanol, 2,3-pentanedione, butanal, (E)-3-hexenoic acid, *β*-pinene and propyl butanoate. In conclusion, these findings suggest that the FWJ with the specific combination of these three LAB strains represents a promising and effective strategy for developing a novel beverage with significantly enhanced the sensory attributes and antioxidant capacity.

## Figures and Tables

**Table 1:** Organic acid composition, TPC, TFC, antioxidant capacity, and sensory evaluation scores of WJ and FWJ prepared via single and mixed LAB fermentations.

Index	WJ	FWJ-SL05	FWJ-SL08	FWJ-002	FWJ-Mix
Lactic acid (mg/mL)	ND	4.16 ± 0.17 ^a^	4.10 ± 0.11 ^a^	4.21 ± 0.13 ^a^	4.22 ± 0.13 ^a^
Acetic acid (mg/mL)	0.49 ± 0.05 ^b^	0.83 ± 0.06 ^a^	0.85 ± 0.06 ^a^	0.81 ± 0.02 ^a^	0.85 ± 0.08 ^a^
Succinic acid (mg/mL)	0.64 ± 0.02 ^c^	1.32 ± 0.06 ^b^	1.31 ± 0.07 ^b^	1.41 ± 0.04 ^ab^	1.46 ± 0.01 ^a^
Pyruvic acid (mg/mL)	0.007 ± 0.001 ^a^	0.010 ± 0.001 ^a^	0.010 ± 0.001 ^a^	0.011 ± 0.001 ^a^	0.010 ± 0.001 ^a^
Tartaric acid (mg/mL)	0.19 ± 0.01 ^b^	0.35 ± 0.07 ^a^	0.38 ± 0.06 ^a^	0.49 ± 0.07 ^a^	0.52 ± 0.09 ^a^
Malic acid (mg/mL)	0.66 ± 0.04 ^a^	0.05 ± 0.01 ^b^	0.05 ± 0.01 ^b^	0.04 ± 0.01 ^b^	0.08 ± 0.02 ^b^
Citric acid (mg/mL)	1.59 ± 0.15 ^a^	0.31 ± 0.02 ^b^	0.37 ± 0.03 ^b^	0.33 ± 0.07 ^b^	0.39 ± 0.09 ^b^
Total organic acid (mg/mL)	3.87 ± 0.19 ^c^	7.37 ± 0.11 ^b^	7.52 ± 0.17 ^b^	7.62 ± 0.21 ^ab^	7.91 ± 0.15 ^a^
TPC (mg GAE/L)	847.23 ± 14.90 ^e^	1121.93 ± 6.67 ^d^	1182.12 ± 11.02 ^c^	1211.7 ± 5.18 ^b^	1421.85 ± 36.68 ^a^
TFC (mg RE/L)	93.25 ± 0.30 ^d^	143.26 ± 1.77 ^c^	140.87 ± 1.10 ^c^	151.95 ± 0.82 ^b^	163.25 ± 0.57 ^a^
ABTS (μmol TE/mL)	1.13 ± 0.05 ^b^	1.45 ± 0.04 ^a^	1.43 ± 0.04 ^a^	1.46 ± 0.07 ^a^	1.54 ± 0.08 ^a^
CURAP (μmol TE/mL)	3.39 ± 0.09 ^c^	3.62 ± 0.07 ^b^	3.65 ± 0.06 ^b^	3.66 ± 0.07 ^b^	3.97 ± 0.11 ^a^
Sensory evaluation score	81.88	88.5	89	90.125	91

Note: WJ refers to wampee juice; FWJ-SL05 refers to fermented WJ with *P. pentosaceus* SL05, FWJ-SL08 with *P. acidilactici* SL08, and FWJ-002 with *L. plantarum* JYLP-002; FWJ-mix refers to fermented WJ with the optimal LAB combination. The values in the same row with different lowercase letters indicate statistically significant differences (*p* < 0.05).

## Data Availability

The original contributions presented in this study are included in the article/Supplementary Materials. Further inquiries can be directed to the corresponding authors.

## Supplementary Materials

The following supporting information can be downloaded at https://www.mdpi.com/article/10.3390/foods14234001/s1. Table S1. Simplex lattice mixture design for mixed fermentation of WJ. Table S2. ANOVA of the simplex lattice mixture design for mixed fermentation of WJ. Table S3. Sensory evaluation table of FWJ. Table S4. Sensory Evaluation of WJ, FWJ-SL05, FWJ-SL08, FWJ-002, and FWJ-Mix samples. Table S5. The key aroma contributors identified via HS-SPME-GC-MS. Table S6. The key aroma contributors identified via HS-GC-IMS. The gene sequences of *P. pentosaceus* SL05 and *P. acidilactici* SL08.
