# Correction: Zhong et al. Genetic Algorithm-Driven Optimization of Mixed-Strain Fermentation for Improving the Physicochemical, Antioxidant, and Sensory Properties of Wampee (*Clausena lansium* (Lour.) Skeels) Juice. *Foods* 2025, *14*, 4001

**DOI:** 10.3390/foods15142485

**Published:** 2026-07-14

**Authors:** Xianquan Zhong, Lin Zhang, Rong Liu, Hua Chen, Zhiheng Zhao, Xiaonuo Li, Kun Cai, Weimin Zhang, Xiaoping Hu, Xue Lin

**Affiliations:** 1 School of Food Science and Engineering, Hainan University, Haikou 570228, China; 23210832000023@hainanu.edu.cn (X.Z.); linzhang@hainanu.edu.cn (L.Z.); chenh330@163.com (H.C.); 21210832000034@hainanu.edu.cn (Z.Z.); qwaslixiao@foxmail.com (X.L.); caikun@hainanu.edu.cn (K.C.); zhwm1979@163.com (W.Z.); 2 Key Laboratory of Food Nutrition and Functional Food of Hainan Province, Haikou 570228, China; 3 Department of Nutrition and Health, Key Laboratory of Functional Dairy, China Agricultural University, Beijing 100083, China; liurong@cau.edu.cn

## Error in Table

In the original publication [1], there was a mistake in Table 2. Analysis of VOCs in WJ and FWJ prepared by single and mixed LAB fermentations by HS-SPME-GC-MS, as published. We noticed that the data in the second column (RI1) and third column (RI2) of Table 2 are incorrect. This mistake occurred at the manuscript submission stage, when we accidentally pasted data from two other columns (Similarity Index, SI; Reverse Similarity Index, RSI) in our draft into Table 2. The corrected Table 2 appears below. The authors state that the scientific conclusions are unaffected. This correction was approved by the Academic Editor. The original publication has also been updated.

## Figures and Tables

**Table 2:** Analysis of VOCs in WJ and FWJ prepared by single and mixed LAB fermentations by HS-SPME-GC-MS.

Category	RI_1_	RI_2_	VOCs	Formula	Content (mg/L)				
					WJ	FWJ-SL05	FWJ-SL08	FWJ-002	FWJ-Mix
Aldehydes	1371	1382	Nonanal	C_9_H_18_O	0.19 ± 0.01 ^a^	0.06 ± 0.00 ^b^	0.03 ± 0.00 ^b^	0.04 ± 0.00 ^b^	0.01 ± 0.00 ^b^
	1661	1662	Benzeneacetaldehyde	C_8_H_8_O	0.02 ± 0.00 ^a^	ND	ND	ND	ND
	1527	1541	Benzaldehyde	C_7_H_6_O	0.42 ± 0.02 ^a^	0.20 ± 0.01 ^b^	0.21 ± 0.01 ^b^	0.19 ± 0.01 ^b^	0.24 ± 0.01 ^b^
Terpenes	1172	1193	D-Limonene	C_10_H_16_	0.20 ± 0.01 ^a^	0.25 ± 0.01 ^a^	0.22 ± 0.01 ^a^	0.29 ± 0.01 ^b^	0.34 ± 0.01 ^b^
	1254	1253	*γ*-Terpinene	C_10_H_16_	0.03 ± 0.00 ^b^	0.04 ± 0.00 ^b^	0.04 ± 0.00 ^b^	0.04 ± 0.00 ^b^	0.19 ± 0.01 ^a^
	1199	1206	*β*-Phellandrene	C_10_H_16_	2.32 ± 0.13 ^b^	3.16 ± 0.13 ^a^	3.18 ± 0.13 ^a^	3.37 ± 0.13 ^a^	3.45 ± 0.14 ^a^
	1004	1020	*α*-Pinene	C_10_H_16_	0.83 ± 0.05 ^d^	1.39 ± 0.06 ^a^	1.01 ± 0.04 ^c^	1.23 ± 0.05 ^b^	1.45 ± 0.06 ^a^
	1141	1160	*α*-Phellandrene	C_10_H_16_	0.68 ± 0.04 ^c^	1.03 ± 0.04 ^b^	1.06 ± 0.04 ^b^	1.06 ± 0.04 ^b^	1.22 ± 0.05 ^a^
	1139	-	(+)-4-Carene	C_10_H_16_	0.10 ± 0.01 ^c^	0.18 ± 0.01 ^b^	0.17 ± 0.01 ^b^	0.19 ± 0.01 ^b^	0.25 ± 0.01 ^a^
Ketones	999	1006	2-Pentanone	C_5_H_10_O	0.36 ± 0.02 ^a^	ND	ND	ND	ND
	961	963	2,3-Butanedione	C_4_H_6_O_2_	ND	0.13 ± 0.01 ^a^	0.12 ± 0.00 ^a^	0.11 ± 0.00 ^a^	0.13 ± 0.01 ^a^
	1341	1318	2-Propanone, 1-hydroxy-	C_3_H_6_O_2_	ND	ND	ND	0.41 ± 0.02 ^a^	0.23 ± 0.01 ^b^
	1300	1296	Acetoin	C_3_H_6_O	0.06 ± 0.00 ^a^	ND	ND	ND	ND
Alcohols	1896	1907	Phenylethyl Alcohol	C_8_H_10_O	0.3 ± 0.02 ^a^	ND	ND	0.1 ± 0.00 ^b^	ND
	912	930	Ethanol	C_2_H_6_O	ND	3.11 ± 0.12 ^a^	3.02 ± 0.12 ^a^	2.94 ± 0.12 ^a^	3.18 ± 0.13 ^a^
	1387	1388	3-Hexen-1-ol, (E)-	C_6_H_12_O	ND	0.15 ± 0.01 ^b^	0.17 ± 0.01 ^b^	0.12 ± 0.00 ^b^	0.56 ± 0.02 ^a^
	1187	1119	2-Propen-1-ol	C_3_H_6_O	ND	0.08 ± 0.00 ^a^	0.07 ± 0.00 ^a^	0.02 ± 0.00 ^b^	0.09 ± 0.00 ^a^
	2197	-	Benzenepropanol, 4-methyl-	C_10_H_14_O	ND	0.32 ± 0.01 ^a^	0.27 ± 0.01 ^a^	0.29 ± 0.01 ^a^	0.36 ± 0.01 ^a^
	1507	1490	1-Hexanol, 2-ethyl-	C_8_H_18_O	0.24 ± 0.01 ^b^	0.35 ± 0.01 ^a^	0.39 ± 0.02 ^a^	0.28 ± 0.01 ^b^	0.38 ± 0.02 ^a^
	1479	1463	1-Heptanol	C_7_H_16_O	1.06 ± 0.06 ^b^	1.17 ± 0.05 ^a^	1.26 ± 0.05 ^a^	1.09 ± 0.04 ^b^	1.13 ± 0.05 ^b^
	1197	1206	1-Butanol, 3-methyl-	C_5_H_12_O	ND	0.36 ± 0.01 ^b^	0.27 ± 0.01 ^c^	0.43 ± 0.02 ^a^	0.34 ± 0.01 ^b^
	1123	1135	1-Butanol	C_4_H_10_O	ND	0.09 ± 0.00 ^b^	0.11 ± 0.00 ^a^	0.14 ± 0.01 ^a^	0.15 ± 0.01 ^a^
	1174	-	3-Buten-2-ol, 2,3-dimethyl-	C_6_H_12_O	0.07 ± 0.00 ^b^	0.11 ± 0.00 ^a^	0.12 ± 0.00 ^a^	0.14 ± 0.01 ^a^	0.13 ± 0.01 ^a^
	1369	-	1-Hexyn-3-ol, 3-methyl-	C_7_H_12_O	ND	ND	ND	ND	0.56 ± 0.02 ^a^
	1687	1683	*α*-Terpineol	C_10_H_18_O	0.31 ± 0.02 ^c^	0.63 ± 0.03 ^b^	0.79 ± 0.03 ^a^	0.66 ± 0.03 ^b^	0.84 ± 0.03 ^a^
Esters	1908	-	Verbenyl angelate, cis-	C_15_H_22_O_2_	0.07 ± 0 ^a^	ND	ND	0.03 ± 0.00 ^b^	0.02 ± 0.00 ^b^
	1373	-	Propyl pyruvate	C_6_H_10_O_3_	ND	0.63 ± 0.03 ^a^	0.55 ± 0.02 ^b^	ND	0.44 ± 0.02 ^c^
	1366	-	Carbonic acid, methyl pentyl ester	C_7_H_14_O_3_	ND	1.97 ± 0.08 ^b^	1.59 ± 0.06 ^c^	0.98 ± 0.04 ^d^	2.43 ± 0.10 ^a^
	1111	-	Propanoic acid, ethenyl ester	C_5_H_8_O2	ND	ND	ND	0.56 ± 0.02 ^a^	0.31 ± 0.01 ^b^
	894	834	Acetic acid, methyl ester	C_3_H_6_O_2_	ND	1.39 ± 0.06 ^c^	1.16 ± 0.05 ^d^	1.98 ± 0.08 ^b^	2.37 ± 0.09 ^a^
	1782	-	Ethyl mandelate	C_10_H_12_O_3_	0.11 ± 0.01 ^b^	ND	ND	ND	0.43 ± 0.02 ^a^
	1494	-	Acetic acid, 2-(methylaminoethyl) ester	C_5_H_11_NO_2_	ND	ND	ND	ND	0.13 ± 0.01 ^a^
	918	-	Acetic acid ethenyl ester	C_4_H_6_O_2_	ND	0.13 ± 0.01 ^a^	0.11 ± 0.00 ^a^	ND	0.13 ± 0.01 ^a^
	1934	-	Oxalic acid, butyl cyclobutyl ester	C_10_H_16_O_4_	0.09 ± 0.01 ^b^	0.42 ± 0.02 ^a^	0.38 ± 0.02 ^a^	0.39 ± 0.02 ^a^	0.42 ± 0.02 ^a^
	1386	-	Isopropyl pyruvate	C_6_H_10_O_3_	ND	0.10 ± 0.00 ^a^	ND	ND	0.11 ± 0.00 ^a^
	1034	1001	Butanoic acid, methyl ester	C_5_H_10_O_2_	ND	0.05 ± 0.00 ^a^	0.03 ± 0.00 ^a^	0.03 ± 0.00 ^a^	0.05 ± 0.00 ^a^
	953	966	n-Propyl acetate	C_5_H_10_O_2_	0.03 ± 0.00 ^a^	0.04 ± 0.00 ^a^	0.05 ± 0.00 ^a^	ND	0.05 ± 0.00 ^a^
	1193	1197	Hexanoic acid, methyl ester	C_7_H_14_O_2_	ND	ND	ND	ND	0.05 ± 0.00 ^a^
	1113	-	Oxalic acid, dipropyl ester	C_8_H_14_O_4_	0.02 ± 0.00 ^a^	ND	ND	ND	ND
Acids	1454	1461	Acetic acid	C_2_H_4_O_2_	3.38 ± 0.19 ^b^	4.34 ± 0.17 ^a^	4.33 ± 0.17 ^a^	4.09 ± 0.16 ^a^	4.47 ± 0.18 ^a^
	1445	-	Acetic acid, diethyl-	C_6_H_12_O_2_	ND	0.04 ± 0.00 ^c^	0.05 ± 0.00 ^b^	0.05 ± 0.00 ^b^	0.08 ± 0.00 ^a^
	2550	2568	Benzeneacetic acid	C_8_H_8_O_2_	ND	0.01 ± 0.00 ^c^	0.03 ± 0.00 ^b^	0.03 ± 0.00 ^b^	0.06 ± 0.00 ^a^
	1728	1734	Pentanoic acid	C_5_H_10_O_2_	ND	0.18 ± 0.01 ^a^	0.12 ± 0.00 ^b^	0.11 ± 0.00 ^b^	0.14 ± 0.01 ^b^
	1768	-	5-Aminovaleric acid	C_5_H_11_NO_2_	ND	0.05 ± 0.00 ^a^	0.08 ± 0.00 ^a^	0.09 ± 0.00 ^a^	0.08 ± 0.00 ^a^
Furans	1674	-	2-Acetyl-2-methyltetrahydrofuran	C_7_H_12_O_2_	0.09 ± 0.01 ^c^	0.16 ± 0.01 ^b^	0.17 ± 0.01 ^b^	0.18 ± 0.01 ^b^	0.31 ± 0.01 ^a^
	1070	-	Furan, 2-methoxy-	C_5_H_6_O_2_	0.13 ± 0.01 ^a^	ND	ND	ND	ND
	955	-	Furan, 2,5-dihydro-3-methyl-	C_5_H_8_O	0.39 ± 0.02 ^a^	ND	ND	ND	ND
	984	-	2-Ethyltetrahydrofuran	C_6_H_12_O	ND	0.08 ± 0.00 ^a^	0.06 ± 0.00 ^a^	0.08 ± 0.00 ^a^	0.09 ± 0.00 ^a^
Hydrocarbon	1000	1000	Decane	C_10_H_22_	0.21 ± 0.01 ^a^	0.22 ± 0.01 ^a^	0.23 ± 0.01 ^a^	ND	ND
	1100	1100	Undecane	C_11_H_24_	0.1 ± 0.01 ^a^	0.1 ± 0.00 ^a^	0.1 ± 0.00 ^a^	ND	ND
	1224	-	Undecane, 4,7-dimethyl-	C_13_H_28_	ND	0.21 ± 0.01 ^b^	0.2 ± 0.01 ^b^	0.21 ± 0.01 ^b^	0.43 ± 0.04 ^a^
	1218	-	Undecane, 3,5-dimethyl-	C_13_H_28_	0.46 ± 0.03 ^a^	ND	ND	ND	0.29 ± 0.01 ^b^
	1200	1200	Dodecane	C_12_H_26_	0.20 ± 0.01 ^a^	0.20 ± 0.01 ^a^	0.20 ± 0.01 ^a^	0.12 ± 0.00 ^b^	0.11 ± 0.00 ^b^
	1329	-	Dodecane, 2,7,10-trimethyl-	C_15_H_32_	ND	0.45 ± 0.02 ^b^	0.41 ± 0.02 ^b^	0.43 ± 0.02 ^b^	0.49 ± 0.02 ^a^
	1210	-	2,3-Dimethyldecane	C_12_H_26_	ND	ND	ND	0.23 ± 0.01 ^a^	ND
	1400	1400	Tetradecane	C_14_H_30_	ND	0.47 ± 0.02 ^b^	0.57 ± 0.02 ^a^	ND	ND
Other	2012	-	Phenol, 4-ethyl-	C_8_H_10_O	ND	0.29 ± 0.01 ^b^	0.28 ± 0.01 ^b^	0.27 ± 0.01 ^b^	0.49 ± 0.02 ^a^
	2011	-	Phenol, 3-ethyl-	C_8_H_10_O	ND	ND	ND	0.13 ± 0.01 ^a^	ND
	2009	2012	Phenol	C_6_H_6_O	0.17 ± 0.01 ^a^	0.13 ± 0.01 ^b^	0.11 ± 0.00 ^b^	0.14 ± 0.01 ^a^	0.18 ± 0.01 ^a^

Note: VOCs is the abbreviation of volatile organic compounds. WJ refers to wampee juice; FWJ-SL05 refers to fermented WJ with *P. pentosaceus* SL05, FWJ-SL08 with *P. acidilactici* SL08, and FWJ-002 with *L. plantarum* JYLP-002; FWJ-mix refers to fermented WJ with the optimal LAB combination. The values in the same row with different lowercase letters indicate statistically significant differences (*p* < 0.05). RI_1_, retention index detected on HP-INNOWax capillary column; RI_2_, retention index recorded in the literature; ND, not detected.
