# Supplementary material for: Genetic Algorithm-Driven Optimization of Mixed-Strain Fermentation for Improving the Physicochemical, Antioxidant, and Sensory Properties of Wampee (Clausena lansium (Lour.) Skeels) Juice
Source: Foods. 2025 Nov 22;14(23):4001. doi: 10.3390/foods14234001 (PMC12692224; doi:10.3390/foods14234001)
Supplement: Supplementary file 1 [file foods-14-04001-s001.zip › foods-3970263-supplementary.pdf]

Table S1. Simplex lattice mixture design for mixed fermentation of WJ

Sample code	*P. pentosaceus* SL05	*P. acidilactici* SL08	*L. plantarum* JYLP-002	TPC (mg GAE/mL)	Lactic acid content (g/L)
	Mass fraction				
1	0	0	1	1.19 ± 0.03^{cd}	4.21 ± 0.07
2	1	0	0	1.12 ± 0.02^{d}	4.17 ± 0.05
3	0.666667	0.166667	0.166667	1.32 ± 0.02^{b}	4.16 ± 0.04
4	0.5	0	0.5	1.30 ± 0.03^{b}	4.20 ± 0.06
5	0.5	0.5	0	1.27 ± 0.01^{b}	4.17 ± 0.03
6	0.333333	0.333333	0.333333	1.41 ± 0.02^{a}	4.19 ± 0.05
7	0.166667	0.666667	0.166667	1.34 ± 0.01^{b}	4.19 ± 0.07
8	0.166667	0.166667	0.666667	1.33 ± 0.02^{b}	4.20 ± 0.04
9	0	0.5	0.5	1.26 ± 0.02^{b}	4.19 ± 0.08
10	0	1	0	1.25 ± 0.04^{bc}	4.19 ± 0.06
11	0	0	1	1.21 ± 0.02^{c}	4.22 ± 0.04
12	1	0	0	1.15 ± 0.02^{d}	4.18 ± 0.07
13	0	1	0	1.18 ± 0.01^{cd}	4.18 ± 0.07

14	0.5	0.5	0	1.23 ± 0.01[c]	4.18 ± 0.02

Note: *P. pentosaceus* represents *Pediococcus pentosaceus*, *P. acidilactici* represents *Pediococcus acidilactici* and *L. plantarum* represents *Lactiplantibacillus plantarum.*

Table S2. ANOVA of the simplex lattice mixture design for mixed fermentation of WJ

Variance source	Model for TPC						Model for lactic acid content					
	Sum of squares	Degree of freedom	Mean square	F-value	*p*-value	Significance	Sum of squares	Degree of freedom	Mean square	F-value	*p*-value	Significance
Model	0.08099	6	0.01350	22.75593	0.00029	significant	0.002756	6	0.000459	4.730	0.0305	significant
Linear mixture	0.00563	2	0.00282	4.74735	0.04979	significant	0.002638	2	0.001319	13.590	0.0039	significant
A*B	0.00778	1	0.00778	13.11804	0.00849	significant	0.000043	1	0.000043	0.440	0.5292	not significant
A*C	0.01427	1	0.01427	24.06374	0.00174	significant	0.000002	1	0.000002	0.017	0.8991	not significant
B*C	0.00229	1	0.00229	3.85869	0.09024	not significant	0.000042	1	0.000042	0.440	0.5294	not significant
A*B*C	0.01040	1	0.01040	17.53337	0.00410	significant	0.000002	1	0.000002	0.020	0.8927	not significant
Lack of Fit	0.00025	3	0.00008	0.08625	0.96392	not significant	0.000479	3	0.000160	3.200	0.1455	not significant

Note: A refers to *P. pentosaceus* SL05, B refers to *P. acidilactici* SL08, and C refers to *L. plantarum* JYLP-002.

Table S3. Sensory evaluation table of FWJ

Index	Evaluation levels			
	**Excellent**	**Good**	**General**	**Worse**
Appearance	Bright appearance and uniform texture	A slightly cloudy appearance	A certainly cloudy appearance with few sediments or suspended matters	A severely cloudy appearance with a lot of sediments or suspended matters
Aroma	A rich, complex, harmonious, aroma with a typical flavor of wampee	An obvious wampee aroma with a monotonous overall-aroma	A light aroma with a slight unpleasant odor	An obviously impure and unpleasant aroma
Taste	Moderate sourness and sweetness with a refreshing and delicate taste	Moderate sourness and sweetness with a slight refreshing taste	General sourness and sweetness with a monotonous overall-taste	An obvious strange taste
Typicality	Unique flavor with a prominent typicality	A flavor with a certain typicality	A flavor with a slight typicality	A flavor without a typicality

Note: excellent represents 100, good represents 75, general represents 50, and worse represents 25.

Table S4. Sensory Evaluation of WJ, FWJ-SL05, FWJ-SL08, FWJ-002 and FWJ-Mix samples

Index	Appearance					Aroma					Taste					Typicality				
	A	B	C	D	E	A	B	C	D	E	A	B	C	D	E	A	B	C	D	E
**Excellent**	6	7	8	7	8	6	8	8	8	8	6	7	7	8	8	6	6	6	8	7
**Good**	2	2	1	1	1	2	2	2	2	2	2	1	1	0	0	2	2	2	1	2
**General**	1	1	1	1	1	2	0	0	0	0	1	1	1	1	1	2	2	2	1	1
**Worse**	1	0	0	1	0	0	0	0	0	0	1	1	1	1	1	0	0	0	0	0

Note: A refers to *WJ group*, B refers to *FWJ-SL05 group*, C refers to *FWJ-SL08 group, D* refers to *FWJ-002 group,* and E refers to *FWJ-Mix group.* The data in each column means the number of evaluators out of ten who vote for each of the four grades-excellent, good, general, and worse-for each indicator.

Table S5. The key aroma contributors identified by HS-SPME-GC-MS

VOCs	Odor descriptions	OAV				
		WJ	FWJ-SL05	FWJ-SL08	FWJ-002	FWJ-Mix
β-Phellandrene	Citrusy, herbal	58.08	79.00	79.50	84.25	86.25
α-Terpineol	Floral, lilac	1.04	2.10	2.63	2.20	2.80
α-Pinene	Fresh, piney, turpentine, resinous	16.56	27.80	20.20	24.60	29.00
α-Phellandrene	Citrusy, minty, slightly peppery	13.53	20.60	21.20	21.20	24.40

Phenol, 4-ethyl-	Smoky, medicinal, leathery, animalic	0.00	29.00	28.00	27.00	49.00
Phenol, 3-ethyl-	Medicinal, phenolic, slightly sweet	0.00	0.00	0.00	13.00	0.00
Phenol	Medicinal, disinfectant, tarry	1.72	1.30	1.10	1.40	1.80
Nonanal	Waxy, citrusy, fatty with floral nuances	19.19	6.00	3.00	4.00	1.00
Hexanoic acid, methyl ester	Fruity, pineapple, sweet, ethereal	0.00	0.00	0.00	0.00	5.00
D-Limonene	Roasty, coffee, nutty, caramelized	4.04	5.00	4.40	5.80	6.80
Butanoic acid, methyl ester	Citrusy, orange, fresh, light	0.00	1.00	0.60	0.60	1.00
Benzeneacetaldehyde	Fruity, apple, pineapple, sweet	5.05	0.00	0.00	0.00	0.00

Benzaldehyde	Honey, sweet, floral with green notes	1.21	0.57	0.60	0.54	0.69
Acetic acid, methyl ester	Almond, maraschino cherry, nutty	0.00	0.70	0.58	0.99	1.19
3-Hexen-1-ol, (E)-	Solvent, ethereal, faintly fruity	0.00	15.00	17.00	12.00	56.00
2-Propanone, 1-hydroxy-	Green, grassy, leafy	0.00	0.00	0.00	8.20	4.60
2-Pentanone	Sweet, caramel, buttery	18.18	0.00	0.00	0.00	0.00
2-Acetyl-2-methyltetr ahydrofuran	Fruity, ethereal	9.09	16.00	17.00	18.00	31.00
2,3-Butanedione	Caramel, sweet, nutty	0.00	130.00	120.00	110.00	130.00
1-Heptanol	Buttery, creamy, caramel	1.77	1.95	2.10	1.82	1.88

1-Butanol, 3-methyl-	Citrusy, green	0.00	1.20	0.90	1.43	1.13
(+)-4-Carene	Malty, whiskey, fusel oil, banana	3.37	6.00	5.67	6.33	8.33

Note: Aromas of volatile compounds are source from https://pubchem.ncbi.nlm.nih.gov/.

Table S6. The key aroma contributors identified by HS-GC-IMS

VOCs	Odor descriptions	rOAV				
		WJ	FWJ-SL05	FWJ-SL08	FWJ-002	FWJ-Mix
Propyl butanoate	pineapple, apple, creamy, rum-like	100.00	100.00	100.00	100.00	100.00
α-Pinene	Sharp, turpentine	76.19	70.50	69.68	90.57	76.60
Ethyl 3-hydroxybutanoate	grape, creamy, dairy	9.23	8.80	9.00	11.35	9.70
Butanal	Green, grassy	340.77	351.60	291.35	372.03	368.71

2-Butoxyethanol	Mildly sweet, solvent	365.28	318.01	347.73	448.70	377.07
β-Pinene	Woody, piney	8.59	32.55	28.40	18.51	29.99
Ethyl 2-methylpropanoate	apple, strawberry, sweet, rum-like	164.93	150.28	143.65	193.50	164.38
Cyclohexanamine	Fishy, amine, pungent	26.06	21.70	19.56	26.60	26.89
Cyclohexanone, 4-methyl-	Minty, camphoraceous, herbal	14.99	13.66	13.02	18.12	15.16
1-Octene	Very faint, waxy	2.27	1.69	2.25	3.08	2.13
Isoprene	Mild rubbery, green, vegetative	0.98	1.80	1.00	1.45	2.61
(E)-2-Octenal	Fatty, green, cucumber	654.44	579.25	603.76	752.05	630.93

2,3-Pentanedione	Buttery, creamy, caramel	1.21	2.02	1.34	1.89	1.11
3-Methyl-3-buten-1-o l	Floral, green	2.43	2.26	2.23	3.11	2.74
3-Mercapto-2-butano ne	Sulfurous, roasted meat, onion	0.46	0.53	0.62	0.48	1.00
3-Methyl-2-butenal	Pungent, caramel, green	231.80	287.26	289.92	330.18	409.91
Butanoic acid	Rancid butter, cheesy, sour	1.85	1.19	1.12	1.47	1.63
3-Hexenoic acid, (E)	Green, fatty	5.65	12.25	11.27	17.81	11.49
Methyl thiocyanate	Pungent, garlic, sulfurous, sharp	1.23	0.86	1.04	1.65	1.20
(E)-2-Heptenal	Citrusy, fatty, almond	455.88	356.12	537.58	681.23	528.39

Note: Aromas of volatile compounds are source from https://pubchem.ncbi.nlm.nih.gov/.

The gene sequences of *P. pentosaceus SL05 and P. acidilactici SL08* are as follows:

P. pentosaceus SL05

cggctagctcctaaaaggttaccccaccggctttgggtgttacaaactctcatggtgtgacgggcggtgtgtacaaggccc
gggaacgtattcaccgcggcatgctgatccgcgattactagcgattccgacttcgtgtaggcgagttgcagcctacagtccg
aactgagaatggttttaagagattagcttaacctcgcggtttcgcgactcgttgtaccatccattgtagcacgtgtgtagccca
ggtcataaggggcatgatgatttgacgtcgtccccaccttcctccggtttgtcaccggcagtctcactagagtgcccaactta
atgctggcaactagtaataagggttgcgctcgttgcgggacttaacccaacatctcacgacacgagctgacgacaaccatg
caccacctgtcattctgtccccgaagggaacctctaatctcttagactgtcagaagatgtcaagacctggtaaggttcttcgc
gtagcttcgaattaaaccacatgctccaccgcttgtgcgggcccccgtcaattcttttgagtttcaaccttgcggtcgtactccc
caggcggattacttaatgcgttagctgcagcactgaagggcggaaaccctccaacacttagtaatcatcgtttacggcatgg
actaccagggtatctaatcctgttcgctacccatgctttcgagcctcagcgtcagttgcagaccagacagccgccttcgcca
ctggtgttcttccatatatctacgcatttcaccgctacacatggagttccactgtcctcttctgcactcaagtctcccagtttccaa
tgcacttcttcggttgagccgaaggctttcacattagacttaaaagaccgcctgcgctcgctttacgcccaataaatccggata
acgcttgccacctacgtattaccgcggctgctggcacgtagttagccgtggctttctggttaaataccgtcactgggtaaaca
gttactcttacccacgttcttctttaacaacagagctttacgagccgaaacccttcttcactcacgcggcgttgctccatcagac
ttgcgtccattgtggaagattccctactgctgcctcccgtaggagtctgggccgtgtctcagtcccaatgtggccgattaccct
ctcaggtcggctacgtatcactgccttggtgagcctttgcctcaccaactagctaatacgccgcgggtccatccagaagtga
tagcagagccatcttttaaaagaaaaccatgcggttttctctgttatacggtattagcatctgtttccaggtgttatcccctgcttc
tgggcaggttacccacgtgttactcacccgttcgccactcacttcgtgttaaaatctcaatcagtacaagtacgtcataatcaat
taacggaagttcgttcgacttgcatgtattaggcacgccgccagcgttcatcctgagccaggatca

P. acidilactici SL08

ggctagctcctaaaaggttaccccaccggctttgggtgttacaaactctcatggtgtgacgggcggtgtgtacaaggcccg
ggaacgtattcaccgcggcatgctgatccgcgattactagcgattccgacttcgtgtaggcgagttgcagcctacagtccga
actgagaatggttttaagagattagctaaacctcgcggtttcgcgactcgttgtaccatccattgtagcacgtgtgtagcccag
gtcataaggggcatgatgatttgacgtcgtccccaccttcctccggtttgtcaccggcagtctcactagagtgcccaactgaa
tgctggcaactagtaataagggttgcgctcgttgcgggacttaacccaacatctcacgacacgagctgacgacaaccatgc
accacctgtcattctgtccccgaagggaacgcctaatctcttaggttggcagaagatgtcaagacctggtaaggttcttcgcg
tagcttcgaattaaaccacatgctccaccgcttgtgcgggcccccgtcaattcttttgagtttcaaccttgcggtcgtactcccc
aggcggattacttaatgcgttagctgcagcactgaagggcggaaaccctccaacacttagtaatcatcgtttacggcatgga
ctaccagggtatctaatcctgttcgctacccatgctttcgagcctcagcgtcagttacagaccagacagccgccttcgccact
ggtgttcttccatatatctacgcatttcaccgctacacatggagttccactgtcctcttctgcactcaagtctcccagtttccaatg
cacttcttcggttgagccgaaggctttcacattagacttaaaagaccgcctgcgctcgctttacgcccaataaatccggataa
cgcttgccacctacgtattaccgcggctgctggcacgtagttagccgtggctttctggttaaataccgtcactgggtgaacag
ttactctcacccacgttcttctttaacaacagagctttacgagccgaaacccttcttcactcacgcggcgttgctccatcagact
tgcgtccattgtggaagattccctactgctgcctcccgtaggagtctgggccgtgtctcagtcccaatgtggccgattaccct
ctcaggtcggctacgcatcatcgccttggtgagccgttacctcaccaactagctaatgcgccgcgggtccatccagaagtg
atagcagagccatcttttaaaagaaaaccaggcggttttctctgttatacggtattagcatctgtttccaggtgttatcccctgctt
ctgggcaggttacccacgtgttactcacccgtccgccactcacttcgtgttaaaatctcattcagtgcaagcacctcataatca
attaacggaagttcgttcgacttgcatgtattaggcacgccgccagcgctcatcctgagccagtatccaa
